# *IDH* Mutation Subgroup Status Associates with Intratumor Heterogeneity and the Tumor Microenvironment in Intrahepatic Cholangiocarcinoma

**DOI:** 10.1002/advs.202101230

**Published:** 2021-07-11

**Authors:** Xiao Xiang, Ziyang Liu, Chong Zhang, Zhao Li, Jie Gao, Changkun Zhang, Qi Cao, Jinghui Cheng, Hengkang Liu, Dingbao Chen, Qian Cheng, Ning Zhang, Ruidong Xue, Fan Bai, Jiye Zhu

**Affiliations:** ^1^ Department of Hepatobiliary Surgery, Peking University People's Hospital Beijing Key Surgical Basic Research Laboratory of Liver Cirrhosis and Liver Cancer Beijing 100044 China; ^2^ Biomedical Pioneering Innovation Center (BIOPIC), School of Life Sciences Peking University Beijing 100871 China; ^3^ Beijing Advanced Innovation Center for Genomics (ICG) Peking University Beijing 100871 China; ^4^ Translational Cancer Research Center Peking University First Hospital Beijing 100034 China

**Keywords:** hepatocellular carcinoma, immunotherapy, isocitrate dehydrogenase‐like tumors, single cell sequencing, subclonal driver, tumor microenvironment

## Abstract

Intrahepatic cholangiocarcinoma (ICC) is highly heterogeneous. Here, the authors perform exome sequencing and bulk RNA sequencing on 73 tumor regions from 14 ICC patients to portray the multi‐faceted intratumor heterogeneity (ITH) landscape of ICC. The authors show that ITH is highly concordant across genomic, transcriptomic, and immune levels. Comparison of these data to 8 published datasets reveals significantly higher degrees of ITH in ICC than hepatocellular carcinoma. Remarkably, the authors find that high‐ITH tumors highly overlap with the *IDH* (isocitrate dehydrogenase)‐mutant subgroup (*IDH*‐SG), comprising of *IDH*‐mutated tumors and *IDH*‐like tumors, that is, those *IDH*‐wildtype tumors that exhibit similar molecular profiles to the *IDH*‐mutated ones. Furthermore, *IDH*‐SG exhibits less T cell infiltration and lower T cell cytotoxicity, indicating a colder tumor microenvironment (TME). The higher ITH and colder TME of *IDH*‐SG are successfully validated by single‐cell RNA sequencing on 17 503 cells from 4 patients. Collectively, the study shows that *IDH* mutant subgroup status, rather than *IDH* mutation alone, is associated with ITH and the TME of ICC tumors. The results highlight that *IDH*‐like patients may also benefit from *IDH* targeted therapies and provide important implications for the diagnosis and treatment of ICC.

## Graphical abstract

Extensive intratumor heterogeneity (ITH) is observed in intrahepatic cholangiocarcinoma across genomic, transcriptomic, and immune levels. isocitrate dehydrogenase (*IDH*) mutation subgroup (*IDH‐*SG) exhibits higher ITH and colder tumor microenvironment. *IDH*‐like patients may also benefit from *IDH* targeted therapies and caution should be made for its therapeutic selection. The study highlights the importance of evaluating *IDH‐*SG status in future clinical management.

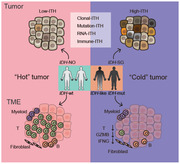

## 1 Introduction

Primary liver cancer (PLC) is the fourth leading cause of cancer‐related mortality worldwide.^[^ ^1^ ^]^ PLC is composed of three major histological subtypes: hepatocellular carcinoma (HCC, ≈80%), intrahepatic cholangiocarcinoma (ICC, ≈15%), and combined hepatocellular and intrahepatic cholangiocarcinoma (cHCC‐ICC, ≈5%).^[^ ^2^ ^]^

ICC is very aggressive, showing a prognosis much worse than HCC and comparable to the most lethal cHCC‐ICC.^[^ ^3^ ^]^ However, the underlying reason has not been comprehensively explored. Currently, surgical resection is the only curative option for localized ICC, but the recurrence rate is high.^[^ ^4^ ^]^ Although the genomic landscape of ICC has been studied,^[^ ^5^, ^6^, ^7^ ^]^ targeted therapies for ICC are still lacking. A deeper understanding of the driver events and evolutionary processes of ICC is needed.

Recently, multi‐region sequencing has revealed the intratumor heterogeneity (ITH) in many cancer types, highlighting a significant molecular barrier for accurate diagnosis and effective treatment.^[^ ^8^, ^9^, ^10^, ^11^, ^12^ ^]^ We and others have characterized the ITH of HCC on multiple dimensions,^[^ ^13^, ^14^, ^15^, ^16^, ^17^ ^]^ showing that ITH is a major obstacle for effective targeted therapies. We also compared the HCC and ICC components in cHCC‐ICC tumors and revealed the relationship between genetic ITH and phenotypic ITH.^[^ ^3^ ^]^ However, the extent of the ITH of ICC and its clinical relevance are not well understood. The ITH of ICC has been studied by multi‐region exome sequencing of cell cultures derived from tumors.^[^ ^18^ ^]^ However, the cell culture system may alter the original tumor clonal substructure and discard the non‐tumorous cells in the tumor microenvironment (TME). Therefore, a comprehensive investigation of the ITH incorporating all the cells is needed. In this study, we performed an integrative genomic analysis of 73 tumor samples from 14 ICC patients, including whole exome sequencing (WES), bulk RNA sequencing (RNA‐seq), and single cell RNA sequencing (scRNA‐seq).

*IDH1* (isocitrate dehydrogenase 1), encoding a metabolic enzyme that catalyzes the oxidative decarboxylation of isocitrate to generate *α*‐ketoglutarate (*α*KG), is a driver gene of ICC (mutational frequency: ≈5–24%)^[^ ^19^, ^20^ ^]^ and many other cancer types, such as low‐grade gliomas (≈70–80%) and acute myeloid leukemia (≈6–10%).^[^ ^21^ ^]^ Missense mutations in the Arg^132^ codon are the most frequent type of mutation in *IDH1*. The resulting mutant *IDH* protein acquires neomorphic enzyme activity and catalyzes the conversion of *α*KG to the oncometabolite 2‐hydroxyglutarate (2HG), which can block cell differentiation by competitively inhibiting *α*KG‐dependent dioxygenases involved in histone and DNA demethylation. Studies in gliomas revealed that the *IDH*‐mutated tumors exhibited a “colder” (non T cell inflamed) TME and a better prognosis in comparison with *IDH*‐wildtype tumors.^[^ ^22^, ^23^, ^24^ ^]^ Both HCC and ICC studies from the Cancer Genome Atlas (TCGA) reported that the *IDH*‐mutant subgroup (*IDH*‐SG) is a distinct molecular subgroup.^[^ ^19^, ^25^, ^26^ ^]^ Here, we showed that *IDH1* mutations were enriched in ICC tumors exhibiting a higher degree of ITH. We found that the transcriptomic profiles of high‐ITH ICC tumors highly overlapped with those of the TCGA‐defined *IDH*‐SG. We further showed that the *IDH*‐mutant/high‐ITH subgroup had less T cell infiltration, which was validated by our scRNA‐seq data. Collectively, our findings link the *IDH*‐SG of ICC with a higher degree of ITH and a “colder” TME, revealing that *IDH*‐SG status shapes ITH and the TME in ICC.

## 2 Results

### 2.1 Patient Cohort and the Genomic Landscape

**Figure 1:**
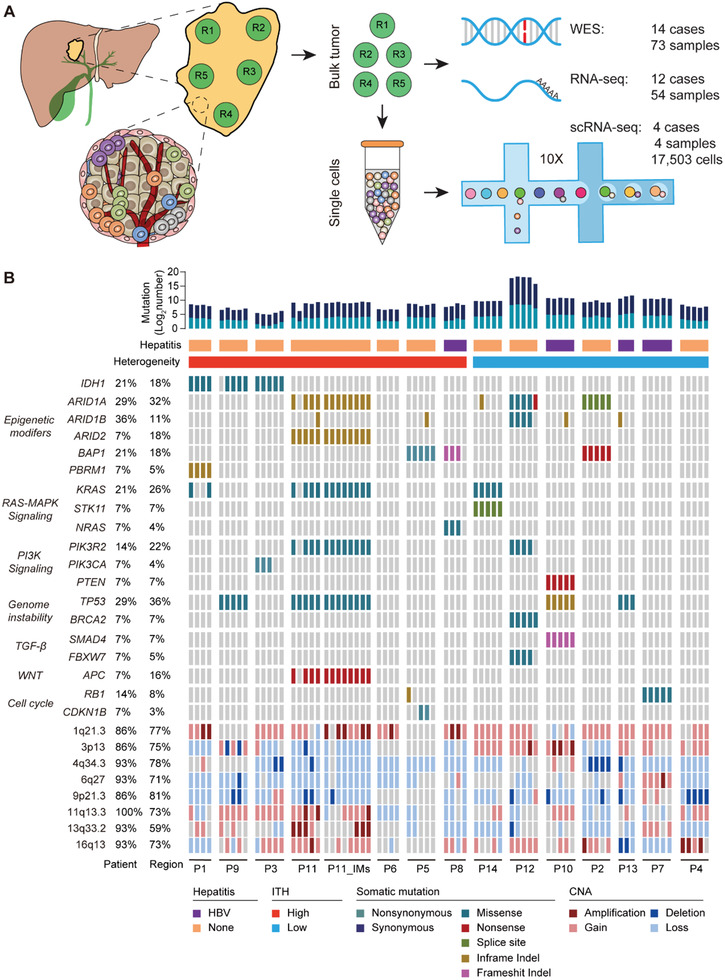
Research strategy and the genomic landscape. A) Research strategy. B) The genomic landscape. Top: number of somatic mutations, viral hepatitis, and ITH group. Middle: mutations of 19 driver genes across all the tumor regions. Driver genes were arranged by signaling pathways. Types of mutations and CNAs are indicated. Bottom: CNAs of 8 recurrent regions: dark red for amplifications, light red for gains, dark blue for deletions, and light blue for losses. Altered frequency based on patient and region is shown on the left.

A total of 87 samples were collected from 14 ICC patients, including 73 tumors and 14 matched non‐tumorous controls. Three to five tumor regions were sampled from each patient per the tumor size (**Figure** **1A** and Table S1, Supporting Information). Immunohistochemical staining (IHC) confirmed the ICC tumor phenotype (Figure S1A, Supporting Information). The average depth of WES was 166× for tumors and 180× for normal controls (Table S2, Supporting Information).

A total of 10 119 somatic mutations were identified, including 9249 (91.4%) point mutations and 870 (8.6%) indels. We examined the mutational status of a set of 19 known driver genes for ICC in our cohort (Figure 1B and Table S3, Supporting Information). *TP53* (29%), *ARID1A* (29%), *KRAS* (21%), and *IDH1* (21%) were among the most frequently mutated genes. Epigenetic regulation was the top affected signaling pathway, altering in 11 patients (78.6%). Many recurrent copy number alterations (CNAs) reported by the ICC_TCGA study^[^ ^19^ ^]^ were also recurrent in our cohort, including gains in 1q21.3 and 11q13.3, as well as losses in 9q21.3 and 4q34.3 (Figure 1B, Figure S1B,S1C, Supporting Information). *FGFR* fusions and HBV integrations were not identified.

### 2.2 DNA Analysis Revealed Extensive ITH in ICC

**Figure 2:**
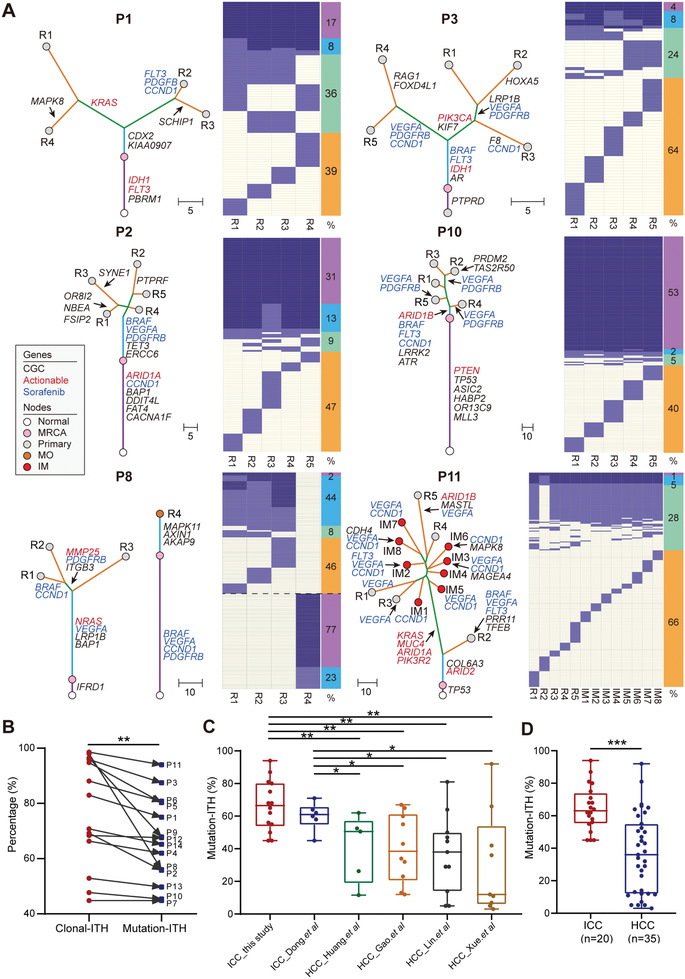
Extensive ITH in ICC observed on the DNA level. A) Heatmaps show the regional distribution of all mutations in six selected patients. Clonal and subclonal mutations are marked in blue and light blue, respectively. The columns next to each heatmap show four categories of mutations and their percentages: trunk clonal mutations (purple); trunk subclonal mutations (sky blue); branch mutations (pale green); and region‐specific mutations (orange). Phylogenetic trees were constructed using a maximum parsimony algorithm based on mutations identified in each patient. The length of each line is proportional to the number of mutations. Mutations in potential driver genes are indicated, including CGC genes (black) and actionable genes (red). Sorafenib‐targeted amplifications are annotated in blue. Patient IDs and region names are labeled in each tree. MRCA, most recent ancestor, IM, intrahepatic metastasis, MO, multiple occurrences. B) Comparison of clonal ITH and mutation‐ITH. ** *P* < 0.01, paired Student's *t* test. C) Comparison of mutation‐ITH between ICC and HCC studies. In the box plots, the lines in the box indicate the median, the boxes indicate the first and third quartiles. * *P* < 0.05, ** *P* < 0.01, Student's *t* test. D) Comparison of the pooled mutation‐ITH of ICC and HCC studies. *** *P* < 0.001, Student's *t* test.

The percentage of trunk mutations, which are present in all tumor regions and located on the trunk of the phylogenetic tree, ranged from 6% to 54% (median 33.3%) (**Figure** **2A**, Figure S2A and Table S4, Supporting Information). Mutation‐ITH was defined as the percentage of non‐trunk mutations to represent the extent of ITH for each patient (Table S4, Supporting Information). For instance, P1 and P3 had relatively high mutation‐ITH (75% and 85%) while P2 and P10 had relatively low mutation‐ITH (56% and 46%). In P8, P8_R4 shared no mutations with other regions, suggesting that P8_R4 had an independent tumor origin.

Previous reports showed that trunk mutations were not all clonal across different tumor regions within a particular patient.^[^ ^27^ ^]^ We calculated the cancer cell fraction (CCF) of mutations in all tumor samples and divided trunk mutations further into subclonal trunk mutations and clonal trunk mutations, with the latter representing the true trunk of a tumor (Figure 2A). Clonal‐ITH was defined as the percentage of both subclonal trunk mutations and non‐trunk mutations. Clonal‐ITH was significantly higher than mutation‐ITH (*P* = 0.0062, paired Student's *t* test), showing that many trunk mutations were actually not clonal (Figure 2B, Figure S2A and Table S4, Supporting Information). 27% of mutations targeting genes in the Cancer Gene Census (CGC) and 29% of mutations targeting actionable genes were clonal (Figure S2B and Table S5, Supporting Information). These results suggest that, sequencing only one tumor region would misinterpret many subclonal trunk mutations as clonal mutations, which may confuse the selection of targeted therapies.

Similarly, we explored recurrent CNAs across these patients and defined CNA‐ITH as the percentage of non‐trunk CNAs. CNA‐ITH varied from 0.9% to 61%, consistent with our observation from mutation‐ITH. In particular, we found that 96% of sorafenib targets, including amplifications in *CCND1*, *BRAF*, *PDGFR*, *VEGFR*, and *FLT3*, were subclonal events, which may explain the low efficacy of sorafenib in ICC (Figure S2B, Supporting Information).^[^ ^4^ ^]^ We observed significant correlation between mutation‐ITH and tumor size (Pearson r squared = 0.83, *P* = 0.00079) as well as CNA‐ITH and tumor size (Pearson r squared = 0.67, *P* = 0.016) (Figure S2C, Supporting Information).

Next, we compared the extent of ITH of our ICC cohort to published multi‐region studies of HCC (Figure 2C). Notably, both ICC_Dong et al. and our cohort exhibited significantly higher mutation‐ITH in comparison with that of four HCC cohorts (all *P <* 0.05, Student's *t* test, Figure 2C).^[^ ^13^, ^28^, ^29^, ^30^ ^]^ This difference was also observed when the mutation‐ITH of the four HCC cohorts and two ICC cohorts were pooled together (*P* < 0.0001, Student's *t* test, Figure 2D and Table S4, Supporting Information).

### 2.3 RNA Analysis Revealed Extensive ITH in ICC

**Figure 3:**
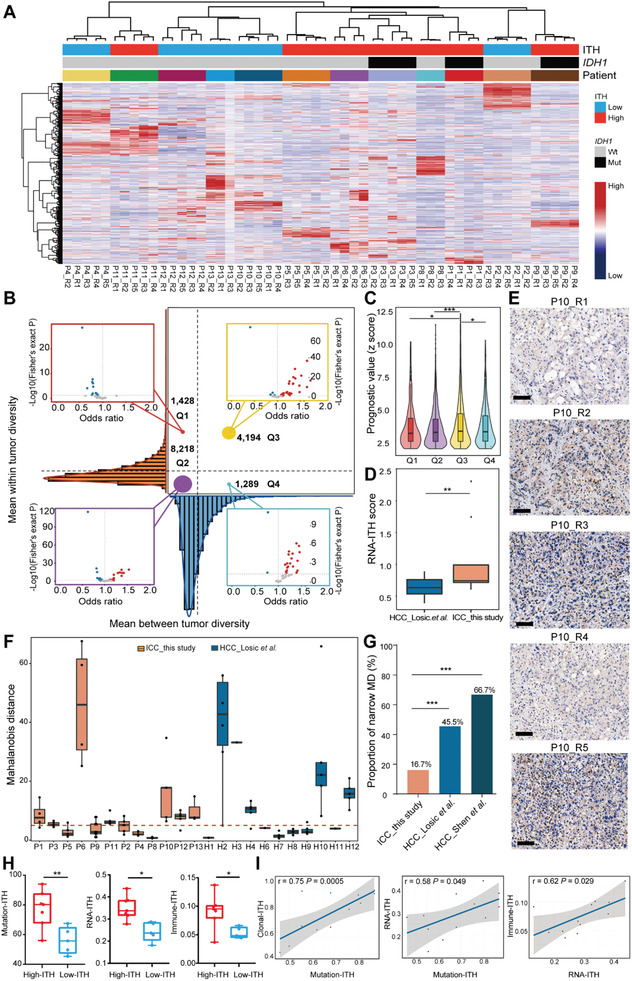
Extensive ITH in ICC observed on the RNA and TME levels. A) Hierarchical clustering of highly variable genes across ICC tumor samples. ITH subgroup, *IDH* mutation, and patient ID are annotated above the heatmap. The Spearman correlation coefficient was used as the distance metric for clustering. B) RNA heterogeneity quadrants for the ICC samples. RNA intratumor (*y*–axis) and intertumor heterogeneity (*x*–axis) are plotted on the axes as density curves. The plot is divided into quadrants by the mean intratumor (dashed horizontal line) and mean intertumor (dashed vertical line) heterogeneity scores. RNA heterogeneity quadrant is indicated for each cancer type as non‐significant (gray), significantly enriched (red; odds ratio > 1), or significantly depleted (blue; odd ratio < 1). Odds ratios are plotted on a natural log scale. Statistical significance was tested with a two‐sided Fisher's exact test. C) Survival association of RNA heterogeneity quadrants across 33 cancer types from the PRECOG database. Boxplots represent median values and 25th and 75th percentiles. The vertical bars span the fifth to 95th percentiles, Student's *t* test. D) Boxplot comparing the RNA‐ITH scores of cases from ICC_this study and HCC_Losic. et al. ** *P* < 0.01, Wilcoxon rank‐sum test. E) IHC of PD‐1 in 5 tumor regions from P10. Scale bar, 100 µm. F) Box plot showing the MD values of immune cells from ICC_this study and HCC_Losic. et al. Red dashed line denotes the MD value of 5. A narrow MD denotes a 0–5 range of MD values for all regions within a tumor. G) Bar plot comparing the proportion of cases with a narrow MD among ICC_this study, HCC_Losic. et al. and HCC_Shen. et al. *** *P* < 0.001, Chi‐square test. H) Box plots comparing the mutation‐ITH, RNA‐ITH, and immune‐ITH of high‐ITH and low‐ITH patients of our cohort. *: *P* < 0.05, **: *P* < 0.01, Student's *t* test. I) Scatter plots showing the Pearson correlations between clonal‐ITH and mutation‐ITH, between mutation‐ITH and RNA‐ITH, and between RNA‐ITH and immune‐ITH.

Unsupervised hierarchical clustering based on the RNA‐seq data showed that regions from the same patients were clustered together, suggesting that inter‐tumor heterogeneity is higher than intra‐tumor heterogeneity in ICC tumors on the transcriptomic level (**Figure** 3A). To quantitatively assess transcriptomic heterogeneity in ICC, we calculated RNA intra‐ and inter‐tumor heterogeneity scores as described.^[^ ^31^ ^]^ Both heterogeneity metrics were split by their mean values, resulting in four RNA heterogeneity quadrants for ICC (Figure 3B). To compare the clinical relevance of these four quadrants, the prognostic scores for each quadrant were calculated based on the prediction of clinical outcomes from genomic profiles (PRECOG) database (Figure 3B,C). The prognostic value of Q3 genes was significantly higher than that of other quadrants (versus Q1, *P* < 0.05; versus Q2, *P* < 0.001; versus Q4, *P* < 0.05, Student's *t* test), showing that the expression of many pan‐cancer prognostic genes was highly heterogeneous either within a particular ICC case or among different ICC cases. This result highlighted the challenge to identify a prognostic biomarker for ICC tumors.

Next, we calculated RNA‐ITH based on the top 100 highly variable genes across tumor regions within each patient. Consistent with our observation on mutation‐ITH, ICC tumors showed significantly higher degrees of RNA‐ITH than that of HCC tumors (HCC_Losic et al.) (*P* = 0.016, Wilcoxon rank‐sum test, Figure 3D and Table S6, Supporting Information).

### 2.4 TME Analysis Revealed Extensive ITH in ICC

We next evaluated the differences of TME among regions from the same patient. We adopted Mahalanobis distance (MD) analysis to represent similarities between random tumor samples within a given patient as described previously.^[^ ^32^ ^]^ MD values were calculated based on two gene lists, including a 35‐gene list for immune cell markers (CD8^+^ T cells, CD4^+^ T cells, regulatory T cells (Tregs), B cells, macrophages, neutrophils, and dendritic cells (DCs)), and a 61‐gene list for immune‐related functional markers (antigen presentation, cell adhesion, co‐inhibitor, co‐stimulator, ligand, cytokine, and exhausted receptor) (Figure S3A,B and Tables S7, S8, Supporting Information). Of note, only 2 of 12 (16.7%, P4 and P8) ICC tumors had a narrow range (0–5) of MD for all regions within the tumor (Figure S3C,D, Supporting Information). These results indicated that 83.3% of ICC patients exhibited a highly heterogeneous TME, which cannot be faithfully evaluated by single region sequencing.

Recent work shows that PD‐1 blockade may serve as a promising therapy for liver cancer.^[^ ^33^ ^]^ We found that PD‐1 mRNA expression varied across different regions from the same tumor (Figure S3B, Supporting Information). For instance, in P10, PD‐1 expression was low in P10_R1 and P10_R4 but high in the other three tumor regions. IHC of PD‐1 further validated this observation (Figure 3E). This result suggests that sequencing a single region from a particular tumor may confuse the therapeutic choice regarding PD‐1 blockade.

We compared the ITH of TME between our ICC cases and published HCC datasets based on the MD values derived from the 35‐gene list for immune cell markers.^[^ ^16^, ^32^ ^]^ We found that the percentage of ICC cases (ICC_this study, 16.7%) exhibiting a narrow MD was significantly lower than the corresponding percentage of HCC cases (versus HCC_Losic et al. 45.5%, *P* < 0.001, versus HCC_Shen et al. 66.7%, *P* < 0.001, Chi‐square test; Figure 3F,G). This result indicated that, compared to HCC, ICC had a much more heterogeneous TME, consistent with our observations on both DNA and RNA levels (Figures 2C,D and 3D). Collectively, these results imply that a higher degree of ITH may contribute to the poorer prognosis of ICC in comparison with HCC.

### 2.5 ITH Was Concordant across Genomic, Transcriptomic, and Immune Levels

To explore the potential interaction of ITH across genomic, transcriptomic, and immune levels, we have investigated the relationship among clonal‐ITH, mutation‐ITH, RNA‐ITH, and immune‐ITH (Table S9, Supporting Information).

In general, we observed a high concordance of ITH across multiple levels. Based on clonal‐ITH, 14 patients from our cohort were divided into high‐ITH and low‐ITH groups. We found that high‐ITH patients showed significantly higher mutation‐ITH (*P* = 0.009, Student's *t* test), RNA‐ITH (*P* = 0.039, Student's *t* test), and immune‐ITH (*P* = 0.021, Student's *t* test) scores than those of low‐ITH patients (Figure 3H). Furthermore, positive correlations were found between clonal‐ITH and mutation‐ITH (Pearson correlation = 0.75, *P* = 0.0005), between mutation‐ITH and RNA‐ITH (Pearson correlation = 0.58, *P* = 0.049), and between RNA‐ITH and immune‐ITH (Pearson correlation = 0.62, *P* = 0.029) (Figure 3I).

### 2.6 Tumors in the *IDH* Mutant Subgroup Exhibited a Higher Degree of ITH

To further explore the clinical relevance of ITH in ICC, we divided patients into two groups based on the median clonal‐ITH value. P1, P3, P5, P6, P8, P9, and P11 were classified into the high‐ITH group, while the other subjects were placed into the low‐ITH group. Interestingly, patients with *IDH1* mutations (P1, P3, and P9) were all in the high‐ITH group (Figure 1B). All three mutations occurred at the *IDH1* ^R132^ hotspot, including *IDH1* ^R132L^ in P1 and *IDH1* ^R132G^ in P3 and P9. *IDH2* mutations were not found in our cohort. We also found that most patients in the high‐ITH group clustered together in the unsupervised clustering of RNA‐seq data, suggesting similar molecular profiles (Figure 3A). Previously, both the HCC_TCGA and ICC_TCGA studies^[^ ^19^, ^25^ ^]^ reported that the *IDH*‐SG is a distinct molecular subgroup. This subgroup is comprised of *IDH*‐mutated (*IDH*‐mut) cases and *IDH*‐mutated‐like cases (*IDH*‐like), that is, those *IDH*‐wild type (*IDH*‐wt) cases that exhibited similar molecular profile to *IDH*‐mut cases. These findings hint that the high‐ITH group of our cohort may correspond to the *IDH*‐SG of TCGA, and *IDH*‐wt cases in our high‐ITH group might be *IDH*‐like cases.

**Figure 4:**
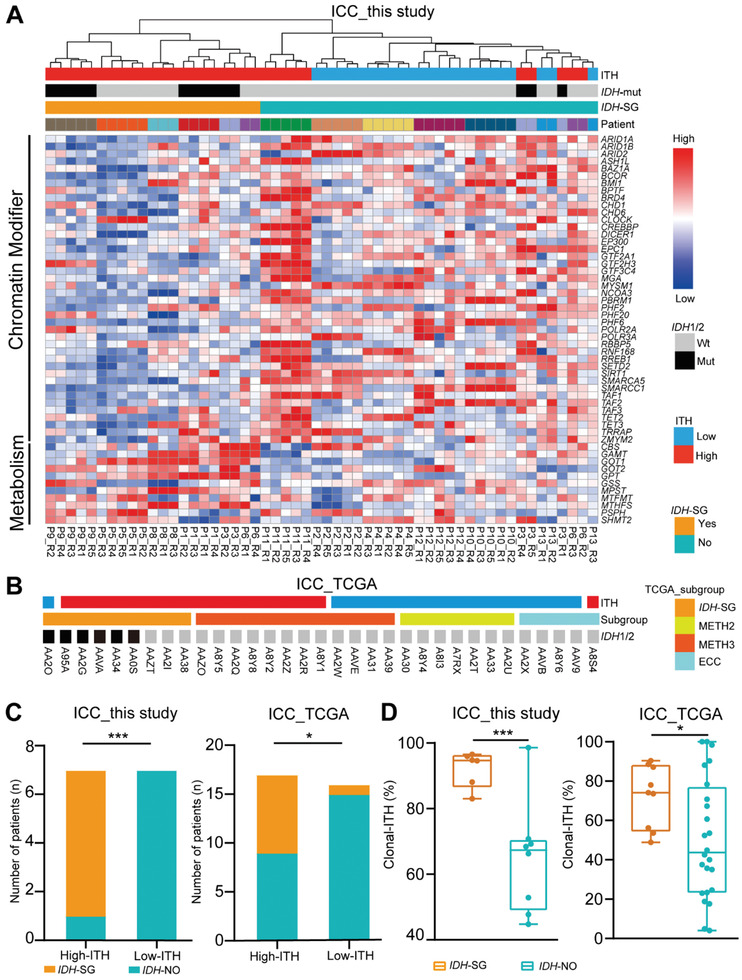
High degree of ITH exhibited by the *IDH* mutation subgroup A) Unsupervised hierarchical clustering of genes related to chromatin modifier and metabolism across ICC samples. ITH status, *IDH* mutation, *IDH*‐SG, and patient ID are annotated. B) 33 cases from ICC_TCGA. ITH status, *IDH* mutation, *IDH*‐SG, and patient ID are annotated. C) Bar plot comparing the number of *IDH*‐SG patients in the groups of high‐ITH and low‐ITH patients in ICC_this study (left) and ICC_TCGA (right). *: *P* < 0.05, ***: *P* < 0.001, Chi‐square test. D) Box plot comparing the clonal‐ITH of *IDH*‐SG and *IDH*‐NO patients in ICC_this study (left) and ICC_TCGA (right). *: *P* < 0.05, ***: *P* < 0.001, Chi‐square test.

According to the ICC_TCGA study, the *IDH*‐SG is enriched for hypermethylation and high metabolic status.^[^ ^19^ ^]^ We performed unsupervised clustering with the 60 genes (Table S10, Supporting Information) reported by the ICC_TCGA study and classified the 54 samples into an *IDH*‐SG (21 samples, 6 cases) and a non‐*IDH*‐mutant subgroup (*IDH*‐NO, 33 samples, 8 cases) (**Figure** **4A**). As expected, the *IDH*‐SG samples were all from the high‐ITH group. Interestingly, although P3 and P6 were from the high‐ITH group, three regions of P3 (P3_R1, P3_R4, and P3_R5) and two regions of P6 (P6_R2 and P6_R3) were classified into the *IDH*‐NO group rather than the *IDH*‐SG group (Figure 4A and Table S11, Supporting Information). Given that patients with one or more tumor regions exhibiting *IDH* signature may all potentially benefit from *IDH* targeted therapies, a patient should be classified as *IDH*‐SG as long as one tumor region from this patient was classified as *IDH*‐SG. Therefore, P3 and P6 were classified as *IDH*‐SG on the patient level. The differences of multiple tumor regions in P3 and P6 demonstrated that although some regions from P3 and P6 shared a relatively similar global transcriptomic profile (Figure 3A), they did show notable differences in *IDH* related genes. Therefore, sequencing only one tumor region cannot faithfully determine the molecular subgroup of some ICC patients. These results demonstrate the extensive ITH of ICC tumors, implying that different regions from the same tumor may be susceptible to different therapies and have different prognoses.

We further used the ICC_TCGA cohort to explore the relationship between ITH degree and *IDH* subgroup status. Since all cases from the ICC_TCGA dataset only had one tumor sample, a direct calculation of ITH from multi‐region sequencing data was not feasible. To infer the ITH of these tumors, we applied a well‐accepted strategy to estimate the clonal substructure from single‐sample tumors.^[^ ^34^, ^35^ ^]^ We inferred the CCF of each mutation by PyClone^[^ ^36^ ^]^ and calculated the percentage of clonal mutations, by the median of which these patients were divided into two groups, “high‐ITH” and “low‐ITH” (Figure 4B and Table S12, Supporting Information).

Both the ICC_TCGA and our cohorts showed that *IDH*‐SG patients were enriched in the high‐ITH group (ICC_TCGA, *P* = 0.0085; ICC_this study, *P* = 0.0012, Chi‐square test; Figure 4C). *IDH*‐SG patients showed clonal‐ITH values significantly higher than those of the *IDH*‐NO subgroup in both our cohort (*P* < 0.001, Student's *t* test) and the ICC_TCGA cohort (*p* = 0.049) (Figure 4D), demonstrating that the *IDH*‐SG of ICC tumors is featured with a high degree of ITH.

Next, we looked into the prognosis of these ICC patients (Figure S4, Supporting Information). Notably, we found that both classifications based on ITH degree and *IDH* subgroup status showed prognostic value in our cohort. By contrast, *IDH*‐mut and *IDH*‐wt patients had no significant prognostic differences, consistent with a previous report on ICC.^[^ ^37^ ^]^ However, classification based on ITH degree and *IDH* subgroup status did not show significant prognostic value in the ICC_TCGA cohort (Table S12, Supporting Information). The potential utility of ITH degree and *IDH* subgroup status as superior markers for prognosis warrants future large cohort studies.

### 2.7 Tumors in the *IDH* Mutation Subgroup Exhibited a “Colder” TME

Previous reports in gliomas showed that *IDH*‐mut and *IDH*‐wt tumors exhibited distinct TMEs.^[^ ^24^ ^]^ To explore whether our *IDH*‐SG and *IDH*‐NO have different TMEs, we deconvolved the cellular composition of 54 ICC tumor regions using xCell (Figure S5A, Supporting Information).^[^ ^38^ ^]^ Notably, in comparison with the *IDH*‐NO samples, *IDH*‐SG samples had fewer CD8^+^ T cells (*P* < 0.001) and more neutrophils (*P* < 0.01, Student's *t* test) (Figure S5B, Supporting Information). Since the *IDH*‐SG cases and high‐ITH cases highly overlapped in our cohort, CD8^+^ T cell deficiency and neutrophil enrichment were also observed in high‐ITH cases (all *P* < 0.01, Student's *t* test; Figure S5C,D, Supporting Information). This result is consistent with a recent study on melanoma showing that high‐ITH tumors had less infiltration of CD8^+^ T cells.^[^ ^39^ ^]^

**Figure 5:**
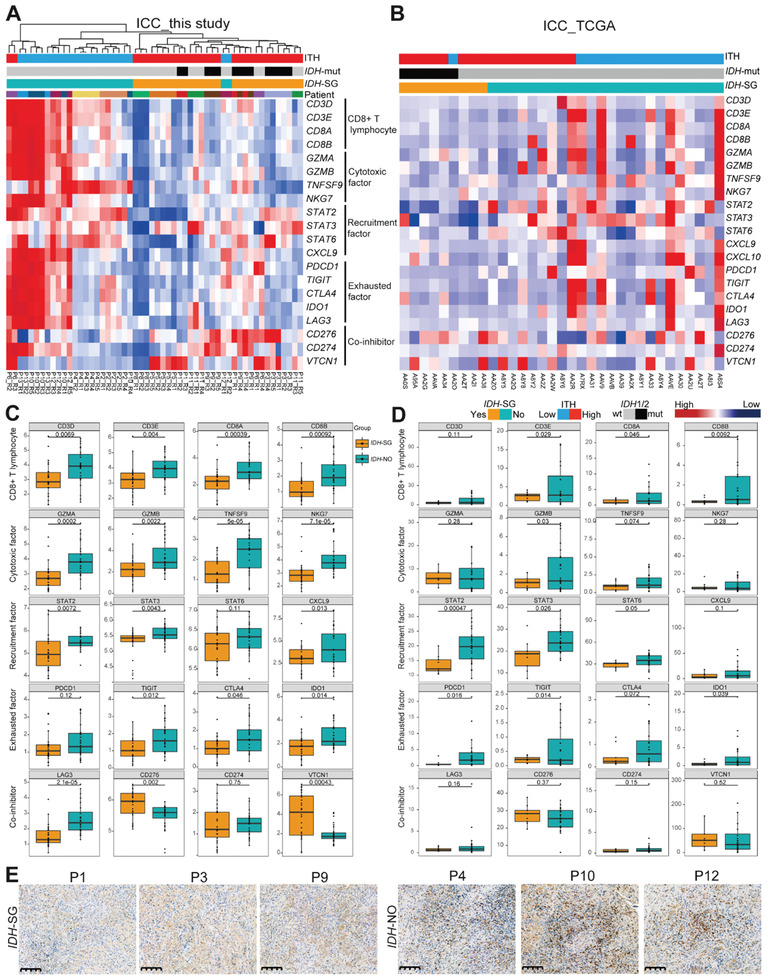
*IDH* mutation subgroup exhibited a distinct tumor microenvironment. A) Unsupervised hierarchical clustering of CD8^+^ T cell‐related markers in our study. B) Heatmap of CD8^+^ T cell‐related markers in the ICC_TCGA study. Boxplots comparing the expression of CD8^+^ T cell‐related markers between *IDH*‐SG and *IDH*‐NO patients C) in our study and D) the ICC_TCGA study. *: *P* < 0.05, **: *P* < 0.05, ***: *P* < 0.001, Student's *t* test. E) IHC of CD8 for six patients. Scale bar, 200 µm.

Neutrophils were scarce among our samples, and we focused on CD8^+^ T cells. We summarized a gene signature comprised of 20 CD8^+^ T cell‐related markers. Unsupervised clustering of samples based on this gene signature showed clear separation of *IDH*‐SG and *IDH*‐NO cases (**Figure** **5A**). Consistent with our observations based on xCell, the *IDH*‐SG cases all exhibited a CD8^+^ T cell‐deficient signature. Concordant results were observed in the ICC_TCGA cohort (Figure 5B). In both the ICC_TCGA and our cohorts, this trend of CD8^+^ T cell deficiency in *IDH*‐SG cases can also be observed across individual genes (Figure 5C,D). Furthermore, IHC of tumor tissue sections confirmed the low level of CD8^+^ T cell infiltration in the *IDH*‐SG cases (Figure 5E). These findings were highly consistent with the observation from gliomas that *IDH*‐mut tumors exhibited a T cell‐deficient TME, whereas *IDH*‐wt tumors showed substantial infiltration of T cells.^[^ ^24^ ^]^

Collectively, our results demonstrate that the *IDH* mutation subgroup status of ICC is associated with distinct characteristics of the TME. Previous immune studies summarize that “hot” tumors are characterized by T cell infiltration and molecular signatures of immune activation, whereas “cold” tumors show prominent features of T cell absence or exclusion.^[^ ^40^ ^]^ Therefore, *IDH*‐SG tumors appear to be cold tumors due to low infiltration of CD8^+^ T cells while *IDH*‐NO tumors seem to be hot tumors as demonstrated by its pronounced infiltration of CD8^+^ T cells.

### 2.8 Single Cell Analysis Revealed Distinct Features of Malignant Cells between *IDH*‐SG and *IDH*‐NO Tumors

**Figure 6:**
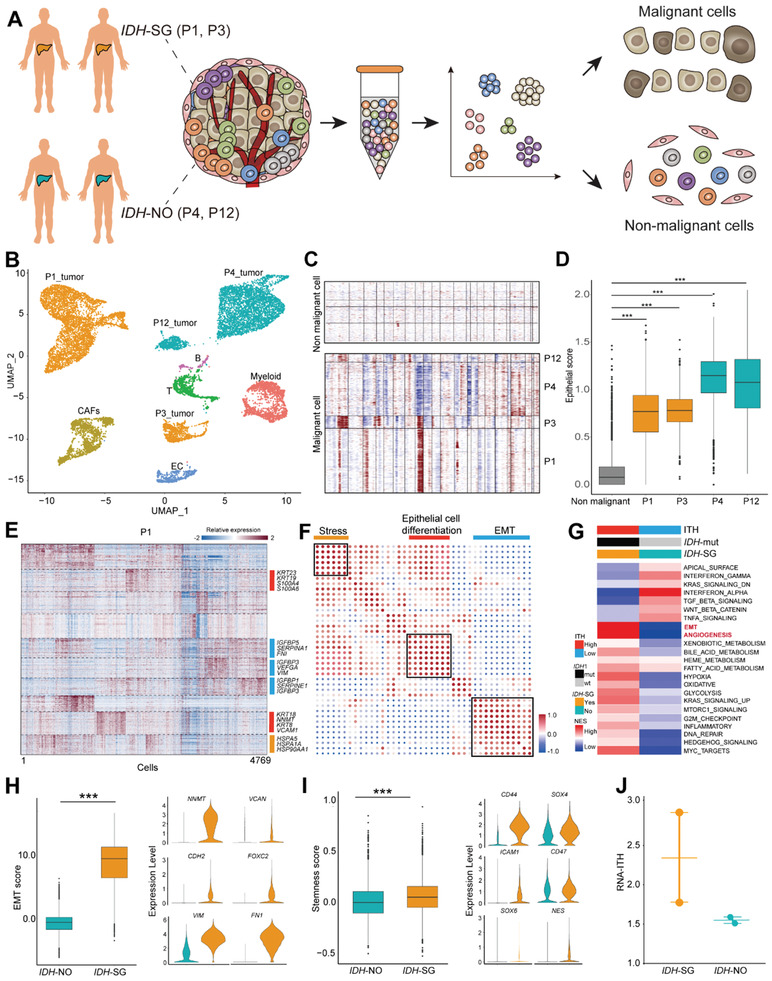
Single‐cell analysis of *IDH*‐SG and *IDH*‐NO tumors. A) Schematic diagram of the scRNA‐seq analysis workflow. B) t‐SNE plots for cell type identification of 15 037 single cells from 4 ICC tumors. C) Large‐scale CNAs of single cells (rows) of 4 ICC tumors. Red, amplifications; blue, deletions. D) Epithelial scores of malignant and non‐malignant cells. In the boxplots, the central rectangles span the first quartile to the third quartile, with the segments inside the rectangle corresponding to the median. The whiskers extend 1.5 times the interquartile range. E) Heatmap showing gene expression programs deciphered from a representative tumor. F) Pearson correlation clustering of 40 intra‐tumor expression programs. The dot size is proportional to the absolute value of the correlation. G) GSVA analysis of malignant cells in *IDH*‐SG and *IDH*‐NO tumors based on the Hallmark Signature from Molecular Signatures Database (MSigDB). The normalized enrichment score (NES) was used to indicate enrichment of the related pathways. H) EMT scores of single cells of *IDH*‐SG and *IDH*‐NO tumors. Violin plots of EMT‐related markers (*NNMT*, *VCAN*, *CDH2*, *FOXC2*, *VIM*, and *FN1*) from *IDH*‐SG and *IDH*‐NO tumors. The width of a violin plot indicates the kernel density of the expression values. All *P* < 0.001, Student's *t* test. I) Stemness scores of single cells of *IDH*‐SG and *IDH*‐NO tumors. Violin plots of stemness‐related markers (*CD44*, *SOX4*, *SOX6*, *ICAM1*, *CD47*, and *NES*) from *IDH*‐SG and *IDH*‐NO tumors. All *P* < 0.001, Student's *t* test. J) Dot plot comparing the RNA‐ITH scores of *IDH*‐SG and *IDH*‐NO tumors.

Single cell sequencing is a powerful tool to dissect the ITH of tumor cells and immune microenvironment.^[^ ^41^, ^42^ ^]^ To further examine the differences between *IDH*‐SG and *IDH*‐NO tumors on the single‐cell level, we selected four representative ICC tumors and performed droplet‐based scRNA‐seq (**Figure** **6A**). After stringent quality control, we retained 15 037 (79.6%) cells for subsequent analysis, comprised of 5129, 3083, 5094, and 1731 cells from P1, P3, P4, and P12, respectively (Table S13, Supporting Information).

We identified six major cell types with t‐distributed stochastic neighbor embedding (t‐SNE) analysis, including T cells, B cells, cancer‐associated fibroblasts (CAFs), endothelial cells (ECs), and epithelial cells (Figure 6B, Figure S6B and Table S14, Supporting Information). We confidently distinguished 10 472 malignant and 4565 nonmalignant cells by complementary approaches.^[^ ^43^, ^44^ ^]^ Briefly, we captured four patient‐specific cell clusters (Figure 6B), from which we inferred large‐scale chromosomal CNAs and examined epithelial marker expression (Figure 6C,D). Non‐negative matrix factorization (NMF) of malignant cells identified three meta‐programs, including epithelial‐mesenchymal transition (EMT), stress, and ribosome (Figure 6E,F and Table S15, Supporting Information). Gene set variation analysis (GSVA) further revealed that malignant cells from *IDH*‐SG tumors up‐regulated pathways such as EMT, angiogenesis, and metabolism (Figure 6G). The EMT and stemness scores of *IDH*‐SG tumor cells were significantly higher than those of *IDH*‐NO tumor cells (Figure 6H,I). Finally, we calculated the RNA‐ITH scores of malignant cells from these cases and found that, two *IDH*‐SG tumors showed a higher degree of RNA‐ITH than two *IDH‐*NO tumors (Figure 6J). This result showed that *IDH*‐SG tumors exhibited a higher degree of ITH on the single cell level, which was consistent with our observation from bulk tumors (Figure 4D).

### 2.9 Single Cell Analysis Revealed Distinct Features of Non‐Malignant Cells between *IDH*‐SG and *IDH*‐NO Tumors

**Figure 7:**
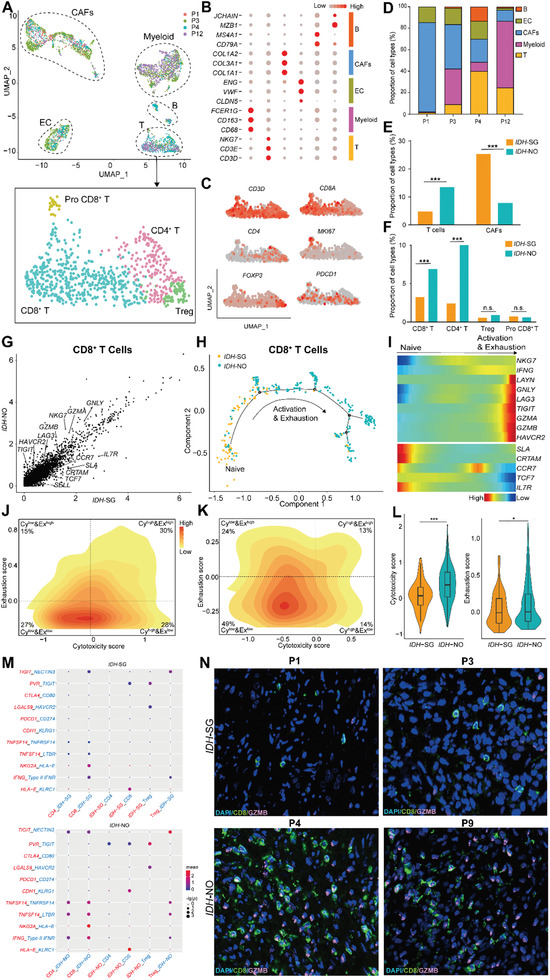
Comparison of non‐malignant cells between *IDH*‐SG and *IDH*‐NO. A) t‐SNE plot of non‐malignant cells from 4 tumors. Cells were annotated based on known lineage‐specific marker genes. 4 subclusters of T cells were highlighted below, including CD4^+^ T cells, CD8^+^ T cells, Tregs, and proliferating T cells. B) Average expression of cell type markers across different clusters. The dot size is proportional to the relative expression level of each gene. C) Expression of T cell markers across different clusters. D) Composition of non‐malignant cells from 4 tumors. E) Bar plot comparing the proportion of T cells and CAFs between *IDH*‐SG and *IDH*‐NO patients. *: *P* < 0.05, ***: *P* < 0.001, Student's *t* test. F) Bar plot comparing the proportion of subclusters of T cells between *IDH*‐SG and *IDH*‐NO patients. *: *P* < 0.05, ***: *P <* 0.001, Student's *t* test. G) Scatterplot showing DEGs in CD8^+^ T cells derived from *IDH*‐SG tumors in comparison with those from *IDH*‐NO tumors. H) The developmental trajectory of CD8^+^ T cells inferred by Monocle2. I) Heatmap showing scaled expression of dynamic genes along the pseudotime. Rows of the heatmap represent genes that show dynamic changes along the pseudotime, and these genes were clustered into two groups according to their expression pattern along the pseudotime. The color scheme represents the z‐score distribution from blue to red. 2D density plot of the cytotoxicity and exhaustion states of CD8^+^ T cells in J) *IDH*‐SG tumors and K) *IDH*‐NO tumors. Cells are partitioned into “high cytotoxicity & high exhaustion” (Cy^high^Ex^high^), “high cytotoxicity & low exhaustion” (Cy^high^Ex^low^), “low cytotoxicity & high exhaustion” (Cy^low^Ex^high^) and “low cytotoxicity & low exhaustion” (Cy^low^Ex^low^) groups. L) Cytotoxicity and exhaustion scores of *IDH*‐SG and *IDH‐*NO tumors. *: *P <* 0.05, ***: *P <* 0.001, Student's *t* test. M) Interaction analysis showing enriched receptor‐ligand pairs in T cell subclusters and malignant cells in *IDH*‐SG tumors (Top) and *IDH*‐NO tumors (down). N) Immunofluorescence for CD8, GZMB, and DAPI for four patients (800×).

Next, we analyzed 4565 non‐malignant cells, including EC, CAFs, myeloid cells, T cells, and B cells (**Figure** **7A**–C, Figure S7 and Table S16, Supporting Information). T cell was further partitioned into CD8^+^, CD4^+^, proliferative and Treg cells.

The proportions of different cell types varied across tumors (Figure 7D). In comparison with *IDH*‐NO tumors, *IDH*‐SG tumors had fewer T cells but more CAFs. *IDH*‐SG tumors had less infiltration of both CD4^+^ (*P* < 0.0001) and CD8^+^ T cells (*P* < 0.0001) (Figure 7E,F). The lack of CD8^+^ T cells in *IDH*‐SG tumors is consistent with our observation on the bulk level (Figure 5A).

Detailed analysis of CD8^+^ T cells further revealed that the quality of CD8^+^ T cells varied between the *IDH*‐SG and *IDH*‐NO tumors. *IDH*‐SG‐derived CD8^+^ T cells showed higher expression of naïve receptors while their counterparts from *IDH*‐NO tumors up‐regulated co‐inhibitory and cytotoxicity receptors (Figure 7G). Consistently, developmental trajectory analysis of CD8^+^ T cells showed that *IDH*‐SG CD8+T cells tended to aggregate at the trunk of the tree, while *IDH*‐NO CD8^+^ T cells accumulated at the branches (Figure 7H,I). Next, we calculated cytotoxicity and exhaustion scores for each cell. The CD8^+^ T cells of *IDH*‐SG tumors were enriched in the Cy^low^Ex^low^ quadrant, suggesting a relatively naïve status. In contrast, in *IDH*‐NO tumors, a large proportion of CD8^+^ T cells were located in the Cy^high^ quadrants, indicating a more active status (Figure 7J,K). After pooling these CD8^+^ T cells together, the *IDH*‐NO group exhibited significantly higher T cell cytotoxicity (*P* < 0.001) and exhaustion (*P* < 0.05) scores in comparison with the *IDH*‐SG group (Figure 7L and Figure S7E, Supporting Information).

These results collectively show that the quantity and quality of CD8^+^ T cells were distinct between the *IDH*‐SG and *IDH*‐NO tumors.

To further explore the relationship between tumor cells and T cells, we analyzed the cell‐cell interaction network (Figure 7M and Table S17, Supporting Information).^[^ ^45^ ^]^ The enrichment of both immune‐activated (*HLA‐E_KLRC1*, *IFNG_IFNR*, *NKG2A_HLA‐E*, *TNFSF14_LTBR*, and *TNFSF14_TNFRSF14*) and immune‐inhibitory (*PDCD1_CD274*, *PVR_TIGIT*, *LGALS9_HAVCR2*, *CTLA4_CD80*, and *CDH1_KLRG1*) interactions in the *IDH*‐NO group corroborated our previous notion that CD8^+^ T cells exhibited an activation‐coupled exhaustion feature. Immunofluorescence staining of P1, P3, P4, and P12 confirmed that *IDH*‐SG tumors displayed less T cell infiltration and lower T cell cytotoxicity in comparison with *IDH*‐NO tumors (Figure 7N).

In summary, analysis of immune cells revealed that *IDH*‐SG tumors displayed less T cell infiltration and lower T cell cytotoxicity in comparison with *IDH*‐NO tumors. Taken together, our scRNA‐seq analysis showed that the *IDH*‐SG and *IDH*‐NO tumors exhibited distinct features both on the tumor cell and immune cell levels, suggesting that patient stratification based on *IDH* mutation subgroup status may be clinically meaningful, indicating distinct therapeutic strategies in the future.

## 3 Discussion

We portrayed the multi‐faceted ITH landscape of ICC and showed that single region sequencing cannot faithfully evaluate the molecular profile of whole ICC tumors. Remarkably, we revealed that ICC tumors exhibited a significantly higher extent of ITH in comparison with HCC, as evidenced by our comparison of mutation‐ITH, RNA‐ITH, and immune‐ITH. High ITH was reported to be associated with poor prognosis in different cancer types.^[^ ^11^ ^]^ Therefore, the higher ITH observed in ICC may provide a critical rationale for its poor prognosis in comparison with HCC.^[^ ^3^ ^]^

Our classification of *IDH*‐SG and *IDH*‐NO showed both significant clinical and biological relevance. Clinically, *IDH*‐SG tumors showed a significantly worse prognosis than *IDH*‐NO tumors. By contrast, *IDH*‐mut and *IDH*‐wt patients had no significant prognostic differences. In fact, the potential prognostic value of *IDH* mutations in ICC tumors has been controversial. *IDH*‐mutated cases were reported to show either improved,^[^ ^46^, ^47^ ^]^ worse,^[^ ^48^ ^]^ or indistinguishable^[^ ^37^ ^]^ overall survival. The inconsistency among these studies may be attributed to small cohort size, biased sample selection, different tumor stages, and diverse postoperative adjuvant treatments. Our result suggested that previous studies may fail to recognize the *IDH*‐like tumors. Our results further showed that patient stratification based on *IDH* mutation subgroup is clinically relevant and may serve as a superior molecular criterion in comparison with mutation status alone. Whether *IDH* mutation subgroup status could serve as a superior marker for prognosis warrants future large cohort studies.

Currently, many targeted therapies have been developed against *IDH* mutations.^[^ ^4^ ^]^ Recently, ivosidenib (AG‐120) showed improved survival in patients with *IDH1*‐mutant ICC.^[^ ^49^ ^]^ We found that a significant proportion of mutations in *IDH1/2* were non‐truncal events across three independent cohorts (ICC_this study, 2/3; ICC_TCGA, 1/3; ICC_Dong et al. 1/1; altogether, 4/7, 57.1%). This result suggests that *IDH1/2* mutations tend to be a subclonal driver and appear at a relatively late stage for ICC. In line with this finding, an ICC study comparing paired primary and recurrent tumors showed that 80% (4/5) of *IDH* mutations were only detected in the recurrent tumors, indicating non‐truncal status.^[^ ^50^ ^]^

The non‐truncal status of *IDH* mutations has important clinical implications: i), sequencing only one tumor region may not accurately genotype *IDH* mutations of some ICC patients. Chances are that some *IDH*‐mut ICC patients are misdiagnosed as *IDH*‐wt ones due to low clonality of *IDH* mutations. In this scenario, whether some *IDH*‐like tumors were actually *IDH*‐mut tumors needs to be confirmed with ultra‐deep targeted sequencing; ii), therapies targeting *IDH* mutations could only inhibit *IDH*‐mut subclones, resulting in a limited therapeutic response. An improved response may be achieved by combinatory therapies targeting both *IDH* mutations and other trunk driver events. iii) *IDH* mutations may be a subclonal driver for ITH. In our cohort, all *IDH*‐SG patients were also in the high‐ITH group, suggesting a potential association between *IDH* subgroup status and the extent of ITH in ICC tumors. Previous multi‐regional studies showed that non‐truncal driver mutations may contribute to a high degree of ITH by preferentially favoring diverse subclonal expansions after establishment of the founding clones.^[^ ^9^, ^12^ ^]^ Therefore, the acquisition of non‐truncal *IDH* mutations during a relatively late stage of tumorigenesis may shape the higher extent of ITH of *IDH*‐SG tumors.

Biologically, *IDH*‐SG tumors displayed less T cell infiltration and lower T cell cytotoxicity in comparison with *IDH*‐NO tumors. By contrast, such differences were not observed when comparing *IDH*‐mut and *IDH*‐wt tumors. Recent studies provided insights into how *IDH* mutations affect the TME of gliomas. Compared to *IDH*‐wt gliomas, *IDH*‐mut tumors showed less infiltration of T cells^[^ ^51^ ^]^ and reduced expression of cytotoxic T‐cell‐associated genes and interferon (IFN)‐*γ*‐inducible chemokines.^[^ ^52^ ^]^ It has been reported that 2HG, the oncometabolite produced by mutant *IDH*, could down‐regulate *STAT1*, thus inhibiting the infiltration of CD8^+^ T cells into tumors.^[^ ^53^ ^]^ Our observations that *IDH*‐SG tumors displayed less T cell infiltration and lower T cell cytotoxicity in comparison with *IDH*‐NO tumors were highly consistent with studies of gliomas, suggesting that *IDH*‐SG tumors are immunologically colder and may be less susceptible to immunotherapies. By contrast, *IDH*‐NO tumors exhibit increased exhausted marker expression and greater T cell infiltration in ICC, and those tumors are considered to be more immunologically hot and more susceptible to immunotherapies. In gliomas, the suppression of T cell accumulation by *IDH* mutation can be reversed by IDH‐C35, a specific inhibitor of mutant *IDH1*.^[^ ^53^ ^]^ This evidence raises the possibility that therapeutic inhibition of mutant *IDH* may convert non‐inflamed cold *IDH*‐SG tumors into hot ones and sensitize them to immunotherapeutic agents.

Therapeutically, *IDH*‐SG tumors may all benefit from targeted therapies against *IDH* mutations due to similar molecular profiles. In particular, since *IDH*‐wt patients from the *IDH*‐SG group, that is, *IDH*‐like patients, exhibit molecular characteristics similar to those of *IDH*‐mut patients, traditional therapeutic choices based simply on *IDH* mutation status may confuse the selection of therapies for these patients. However, in the patient stratification of *IDH*‐mut versus *IDH*‐wt, those *IDH*‐like patients cannot reap the benefit of *IDH* targeted therapies. *IDH*‐like patients may benefit from targeted therapies against *IDH* mutations while being less sensitive to immunotherapies as they are immunologically colder in comparison with other *IDH*‐wt patients. However, in the patient stratification of *IDH*‐mut versus *IDH*‐wt, those *IDH*‐like patients cannot reap the benefit of *IDH* targeted therapies. Therefore, our patient stratification based on *IDH*‐SG and *IDH*‐NO tumors provided a different perspective on the diagnosis and treatment of ICC tumors.

Previous studies showed that markedly elevated levels of serum 2HG can be detected in *IDH*‐mutated ICC and gliomas.^[^ ^21^, ^54^, ^55^ ^]^ Our results hint that *IDH*‐SG tumors may show significantly higher level of 2HG in comparison with *IDH*‐NO tumors. Should this be validated in a future large‐cohort study, detecting 2HG will help identify *IDH*‐SG tumors in the clinical context. Importantly, whether serum 2HG could help identify minor *IDH*‐mutated subclones in certain ICC tumors that might be missed by single region sequencing in clinical setting is also worth investigating. In addition, our results also implied that serum 2HG may serve as a potential biomarker for the ITH degree of ICC tumors.

Altogether, our study, for the first time, shows that *IDH* mutation subgroup status shapes the ITH and TME of ICC tumors. Our results underscore the importance of evaluating the *IDH* mutation subgroup status of ICC tumors in future clinical practice and provide critical information on the diagnosis, prognosis, and treatment of ICC tumors.

## 4 Experimental Section

### Patient Cohort

Fourteen patients diagnosed with ICC were enrolled in this study after approved by the Ethics Committee of Peking University People's Hospital (2019PHB231‐01). All patients in this study provided written informed consent for sample collection and data analyses. None of the patients was treated with chemotherapy or radiation prior to tumor resection. The clinical characteristics of these patients are summarized in Table S1, Supporting Information. Immediately after the surgery, three to five tumor regions and an adjacent non‐tumorous liver tissue were collected from each patient per the tumor size. For each sample region, tissue samples were bisected with half of the tissue sent for sequencing experiments and the other half for pathologic examination. All tumor samples were isolated from viable regions and confirmed to contain more than 70% tumor cells, and non‐tumorous samples were confirmed to be free of tumor cells.

DNA was successfully isolated and quality‐checked for 73 tumor regions from14 cases. RNA was successfully isolated and quality checked for 54 tumor regions from 12 of the 14 cases. Among the 14 patients, scRNA‐seq was performed on 4 cases. Fresh tumor tissues were collected and sent for tissue digestion.

Full details of the methods used are provided in Supporting Information.

## Conflict of Interest

The authors declare no conflict of interest.

## Author Contributions

X.X., Z.Y.L., C.Z., and Z.L. contributed equally to this work. X.X., R.X., F.B., and J.Z. conceived the project. X.X., Z.L., J.G., and C.K.Z. collected the samples. X.X. and R.X. performed experiments. X.X., Z.Y.L., C.Z., Q.C., J.C., and H.L. analyzed the data. R.X., X.X., Z.Y.L., C.Z., N.Z., and F.B. intepreted the data. Z.L., and D.B.C. performed pathological analysis. Q.C., and J.Z. provided clinical information. R.X., and X.X. wrote the manuscript with help from all authors. R.X., F.B., and J.Z. supervised the project.

## Supporting information

Supporting Information

Supplemental Table 1

## Data Availability

The raw sequence data have been deposited in the Genome Sequence Archive in Beijing Institute of Genomics (BIG) Data Center, under accession number HRA000275 (https://bigd.big.ac.cn/gsa‐human/).
