# Supplementary material for: IDH Mutation Subgroup Status Associates with Intratumor Heterogeneity and the Tumor Microenvironment in Intrahepatic Cholangiocarcinoma
Source: Adv Sci (Weinh). 2021 Jul 11;8(17):2101230. doi: 10.1002/advs.202101230 (PMC8425914; doi:10.1002/advs.202101230)
Supplement: Supplementary file 1 — Supporting Information [file ADVS-8-2101230-s001.pdf]

© 2021 Wiley-VCH GmbH

ADVANCED SCIENCE
Open Access

Supporting Information

for *Adv. Sci.,* DOI: 10.1002/advs.202101230

IDH mutation subgroup status associates with intratumor heterogeneity and the tumor microenvironment in intrahepatic cholangiocarcinoma

Xiao Xiang, Ziyang Liu, Chong Zhang, Zhao Li, Jie Gao, Changkun Zhang, Qi Cao, Jinghui Cheng, Hengkang Liu, Dingbao Chen, Qian Cheng, Ning Zhang, Ruidong Xue, Fan Bai*, and Jiye Zhu**

Supplemental methods

DNA isolation and exome sequencing

Tumor and adjacent non-tumorous liver samples were stored as fresh frozen tissue. DNA was isolated using the DNeasy Blood & Tissue Kit (Qiagen, Cat# 69504). The DNA concentration was measured using Qubit 3.0 (Invitrogen). Next, the DNA size was checked using Fragment Analyzer (Advanced Analytical Technologies). 500 ng to 1 mg of DNA was sheared into fragments of approximately 300 bp using Covaris S2 ultrasonicator (Covaris). The library was constructed using the NEBNext Ultra DNA Library Prep Kit for Illumina (New England Biolabs) according to the manufacturer's protocol. Exome capturing was performed using SureSelectXT Human All Exon V6 (target size 60 Mb, Agilent, Santa Clara, CA). Captured genomic DNA was sequenced using paired-end Illumina NovaSeq (Illumina, San Diego, CA) with 150 base pairs (bp) read length, and at least 20 gigabases per sample were produced.

The adapter sequences in the raw data were removed using Cutadapt (v1.14).[1] The remaining adapter-trimmed reads were assessed using two criteria: 1) if the total number of bases with base quality scores less than 20 exceeded half of the read length, and 2) if the total number of undetermined base N in the read exceeded 10% of the read length. If either read of a read pair met one of the above criteria, the read pair was removed. All high-quality reads were subjected to gapped alignment with the UCSC human reference genome (hg19) using the Burrows-Wheeler Aligner (version 0.7.15) with default parameters.[2] The resulting BAM files were sorted, and duplicated reads were marked using Picard tools (v2.11.0) (http://broadinstitute.github.io/picard/). Next, the reads were realigned to the genome and base quality scores were recalibrated using the Genome Analysis Toolkit (GATK 3.8.0) to produce analysis-ready BAM files. [3]

Somatic mutation

Point mutations were called with MuTect (v1.1.4).[4] Small insertions and deletions (Indels) were called using Strelka (v2.8.4).[5] To ensure accurate calling, a series of filtering criteria were applied to the variant candidates. A mutation call was kept if: 1) the site had at least 10X total coverage and at least 3X mutation coverage in the tumor sample; 2) at least 10X total coverage and at most 1X mutation coverage in the matched normal sample. Only indel calls marked with 'PASS' in the VCF file were kept. All high-quality variants were then annotated with SnpEff 3.0.[6]

Copy number alteration

Sequenza (version 2.1.2) was used to analyze copy number alterations (CNAs) while considering both ploidy and cellularity.[7] Specifically, the BAM files from the normal control and tumor samples of each patient were input into the main program. For each heterozygous site, the depth ratio was calculated and then normalized based on the GC content bias of the genome. To obtain segmented copy number data, the following parameters were used: breaks.method='full', gamma=40, kmin=5. Sex chromosomes were excluded from this analysis. We used Genomic Identification of Significant Targets in Cancer (GISTIC)[8] to identify recurrent CNAs across the cohort. For each sample, we first filtered out segments with a total length of less than 500 kb.

Mutation heatmap and phylogenetic tree

The mutation matrix was represented with the binary value of 0/1 indicating the absence/presence of mutations. We applied PyClone (version 0.13.0) to calculate the cancer cell fraction (CCF) value for each identified mutation.[9-10] Mutations with CCF > 0.7 and shared by all tumor regions were considered clonal mutations, and the rest were considered to be subclonal mutations. To reveal the clonal relationship of the tumor samples from each patient, phylogenetic trees were constructed using MEGA5.[11] Sequences of 20 bp in length surrounding the non-silent mutations, as

well as all mutations, were extracted to construct the phylogenetic trees of each patient based on the maximum-parsimony algorithm. All phylogenetic trees were further optimized using Adobe Illustrator. Three categories of potential driver events were labeled on the tree: genes in black were those documented by the Cancer Gene Census (CGC) (https://cancer.sanger.ac.uk/census); genes in red were actionable genes extracted from the DrugBank database (https://www.drugbank.ca/); genes in blue were sorafenib-targeted amplifications, including *BRAF*, *CCND1*, *FLT3*, *VEGFA*, and *PDGFRB*.

Mutation-ITH, clonal-ITH and CNA-ITH

For the multi-region samples (R1, R2, R3, R4, R5) of each patient, we calculated the proportion of non-shared mutations relative to the total number of mutations as the mutation-ITH index by the following formula:

$$\mathrm{Mutation} - \mathrm{ITH} = 1 - \frac{\mathrm{R1} \cap \mathrm{R2} \cap \mathrm{R3} \cap \mathrm{R4} \cap \mathrm{R5}}{\mathrm{R1} \cup \mathrm{R2} \cup \mathrm{R3} \cup \mathrm{R4} \cup \mathrm{R5}}$$

Genes with CCF > 0.7 and shared by all tumor regions were considered clonal mutations, and the remaining mutations were considered as subclonal mutations. We calculated the proportion of clonal mutations relative to the total number of mutations as the clonal ITH by the following formula:

$$\mathrm{Clonal} - \mathrm{ITH} = 1 - \frac{\mathrm{A}}{\mathrm{R1} \cup \mathrm{R2} \cup \mathrm{R3} \cup \mathrm{R4} \cup \mathrm{R5}}$$

A: Number of clonal mutations

We next evaluated the heterogeneity of CNAs for ICC patients. We used GISTIC to identify recurrent CNAs across the cohort. Through comparisons of different samples in each case, amplification and deletion CNAs were classified into shared and non-shared categories. For the multi-region samples (R1, R2, R3, R4, R5) of each patient, we calculated the proportion of non-shared CNAs relative to the total number

of CNAs as the CNA-ITH index by the following formula:

$$\mathrm{CNA-ITH} = 1 - \frac{\mathrm{B}}{\mathrm{R1} \cup \mathrm{R2} \cup \mathrm{R3} \cup \mathrm{R4} \cup \mathrm{R5}}$$

B: Number of shared CNAs

RNA isolation and sequencing

50–100 mg of tissue samples was ground in a liquid-nitrogen-cooled mortar, after which 1 mL of TRIzol™ Reagent (Invitrogen, Cat# 12183555) was immediately added to further homogenize the sample. Total RNA was isolated using the RNeasy Mini Kit (Qiagen, Cat# 74106), after which mRNA was enriched, fragmented, reversed-transcribed into cDNA, and subjected to end-repair and adapter ligation processes. RNA-seq libraries were constructed using the NEBNext Ultra RNA Library Prep Kit (New England Biolabs) according to the manufacturer's protocol. Finally, the library (2x 150-bp paired-end reads) was quality-checked and sequenced with Illumina Novaseq 6000 system (Illumina).

Qualified reads were obtained after removing raw reads with adapters or of low quality and then aligned to the human genome (hg19) using STAR (version 2.6.1c).[12] The fragments per kilobase of exon per million mapped reads (FPKM) values and gene count values were computed using RSEM (version 1.3.1). Differentially expressed genes (DEGs) between *IDH*-SG/*IDH*-NO tumors were identified by DESeq2 using the gene count values as input.[13] Genes with absolute fold change ≥ 1 and p value ≤ 0.01) were considered as DEGs and were used to perform pathway enrichment analysis.

Hierarchical clustering of ICC tumor samples

For all tumor samples with available RNA-seq data, FPKM values generated by RSEM were used, and highly variable genes (HVGs) across samples were selected to

perform hierarchical clustering. Briefly, we removed genes expressed in less than 10% of all available samples and log-transformed the remaining genes. Next, we calculated the coefficient of variation (CV) value for each gene and selected those with CV values greater than 0.8 as HVGs. Euclidean distance was used as the distance metric, and ward. D2 was set as the clustering method to cluster samples.

RNA-ITH on the gene level

To systematically analyze transcriptomic ITH using gene expression data, we adopted the method introduced by a recent study on non-small cell lung cancer.[14] Briefly, we defined two metrics, RNA intertumor heterogeneity (RNA-InterTH) and RNA intratumor heterogeneity (RNA-InterTH, denotes as RNA-ITH in keeping with other ITH metrics), to quantify heterogeneities across different patients and across regions of the same patient, respectively.

To derive the RNA-ITH score, the standard deviation (SD) of the expression values for each gene across the tumor regions of a given patient was calculated to yield a gene-specific, patient-specific measure of RNA-ITH. This process was repeated for all genes, followed by all tumors, generating a gene-by-patient matrix. RNA-ITH scores were summarized as the average value per gene across all tumors in the cohort by the following formula:

$$\mathrm{RNA}-\mathrm{ITH}=\frac{\sum_{i=1}^{i=n}\sigma_{g_i}}{n}$$

Where $\sigma_{g_i}$is the standard deviation of the ith gene across all regions from the same patient and n is the number of genes retained.

To derive the RNA-InterTH score, we randomly sampled one region per patient and calculated the SD for each gene across the resulting single-biopsy cohort. This process was repeated 10,000 times and the average score across iterations was calculated for each gene to yield the RNA-InterTH score.

Finally, we split the RNA-InterTH and RNA-ITH scores by their corresponding mean values to generate the RNA heterogeneity quadrants. To quantitively compare the clinical relevance of these four quadrants, the prognostic scores for each quadrant were calculated based on the Prediction of Clinical Outcomes from Genomic Profiles (PRECOG) database (http://precog.stanford.edu)

RNA-ITH on the patient level

To systematically compare the gene expression heterogeneity between ICC and HCC datasets, we calculated the RNA-ITH score for each patient. We first combined the gene expression matrix of ICC and HCC and kept genes expressed across all ICC and HCC samples. Next, we calculated the CV values for each gene across all tumor regions for each patient, yielding a patient-specific, gene-specific CV matrix. Next, for each patient, we selected the top 100 genes with the highest CV values and took the average as the RNA-ITH score for that specific patient.

Mahalanobis distance

We used the Mahalanobis distance (MD) to assess ITH on the immune level as described.[15] MD is the distance from each region to the centroid in a multi-dimensional space by taking both variances and covariances into account, which was calculated by the following formula:

$$MD = \sqrt{(\chi_r - \mu_R)^T S^{-1} (\chi_r - \mu_R)}$$

where χ_r is the quantification level of immune markers of a specific region r in tumor R; $\mu_R$is the mean quantification level of immune markers in tumor R, and S is the covariance matrix of all markers.

The tumor immune microenvironment was considered similar across different regions if the distance of each region within a tumor was close to 0. If the distribution of MD

values of a given tumor fell within the range of 0-5, then the MD of this tumor was considered to be narrow, indicating that the immune microenvironment was highly similar across all regions of the given tumor.

Immune-ITH

To quantify the ITH of different regions on the immune level, we defined the immune-ITH score as previously described.[16] For patients with ⩾ 2 tumor sectors, we first calculated the pairwise Spearman's correlation between different tumor sectors from the same patient, then the immune-ITH was defined as:

$$\mathrm{Immune} - \mathrm{ITH} = 1 - \mathrm{Median}(1 - \frac{6\sum_{i=1}^{i=N} d_i^2}{NN^2})$$

Where N is the number of data pairs and d_i is the rank difference of the ith data pair.

Inference of immune cell fractions from bulk tumor

The cellular composition of infiltrated immune cells was inferred by xCell.[17] The differences in immune cell enrichment scores between high-ITH/low-ITH patients and *IDH*-SG/*IDH*-NO patients were compared using Student's *t* test. Nineteen $CD8^+$ T cell-related markers, including 4 genes determining cell identity (*CD3D*, *CD3E*, *CD8A*, and *CD8B*), 4 cytotoxic factors (*GZMA*, *GAMB*, *NKG7*, and *TNFSF9*), 4 recruitment factors (*STAT2*, *STAT6*, *CXCL9*, and *CXCL10*), 4 exhausted factors (*PDCD1*, *TIGIT*, *LAG3*, and *IDO1*) and 3 co-inhibitors (*CD276*, *CD274*, and *VTCN1*) were used to cluster the samples. Unsupervised hierarchical clustering was performed with the ward. D2 method using Euclidean distance as the distance metric.

Classification of *IDH* mutation subgroup

Classification of *IDH*-mutant subgroup (*IDH*-SG) and a non-*IDH*-mutant subgroup (*IDH*-NO) was performed both on the sample level and the patient level. For each tumor sample, unsupervised clustering based on the expression of 60 *IDH* signature

genes (**Table S10**) can robustly classify the samples into *IDH*-SG and *IDH*-NO groups. *IDH*-SG can be further confirmed with the presence of *IDH* mutated cases. Given that patients with one or more tumor regions exhibiting *IDH* signature may all potentially benefit from *IDH* targeted therapies, a patient should be classified as *IDH*-SG as long as one tumor region from this patient was classified as *IDH*-SG.

Single cell suspension

To avoid the sampling bias of single tumor regions, the single cell suspension was prepared from the mixture of multiple tumor regions from the same patient. All tumor samples from the same patient were mixed and then minced on ice to generate smaller pieces (less than 1 mm^3) and transferred to 10 mL digestion medium containing 0.2% collagenase I/II (ThermoFisher Scientific), DNAse I (Sigma) and 25 units dispase (Invitrogen) in DMEM (ThermoFisher Scientific). Samples were incubated for 15 min at 37 °C, with manual shaking every 5 min. Next, 30 mL ice-cold PBS, pH 7.4, (ThermoFisher Scientific) containing 2% fetal bovine serum (ThermoFisher Scientific) was added, and the samples were filtered using a 40-µm nylon mesh (ThermoFisher Scientific). Following centrifugation at 120 × *g* for 5 min at 4 °C, the supernatant was decanted and discarded, and the cell pellet was resuspended in 2 mL red blood cell lysis buffer and transferred to a 2-mL DNA low bind tube. Next, 10 µL of this cell suspension was counted using an automated cell counter (Luna) to determine the concentration of live cells. Throughout the dissociation procedure, cells were maintained on ice whenever possible, and the entire procedure was completed in less than 1 h (typically ~45 min) to avoid dissociation-associated artifacts. Sample viability and cell concentration was assessed for each sample.

Single cell cDNA library preparation and sequencing

Single-cell cDNA library was prepared with the 10x Genomics Single Cell 3’ v3 Reagent Kit according to the manufacturer’s protocol. cDNA was obtained after GEM generation and barcoding, followed by the GEM RT reaction and cleanup steps. The

samples were then run on a Bioanalyzer (Agilent Technologies) to determine the cDNA concentration. cDNA libraries were prepared as recommended by the 10x Genomics v3 user guide with appropriate modifications to the PCR cycles based on the calculated cDNA concentration. The resulting libraries were sequenced on an Illumina NovaSeq 6000 system. For sample preparation on the 10x Genomics platform, the Chromium Single Cell 30 Library and Gel Bead Kit v3 (PN-120237), Chromium Single Cell 30 Chip kit v3 (PN-120236) and Chromium i7 Multiplex Kit (PN-120262) were used.

Processing of scRNA-seq data

Raw sequencing reads were aligned to reference genome hg38 using CellRanger (version 3.0.2) to generate a raw gene expression matrix for each sample. Downstream analysis was performed using the Seurat[18] R package (version 3.1.5). Briefly, we removed genes expressed in less than 0.1% of all cells and removed cells with total expressed genes of less than 200 or more than 7,500. Cells with proportions of expressed mitochondrial genes of more than 30% were also removed. All downstream analysis was performed following the instructions proposed at https://satijalab.org/seurat. The cell clusters in the resulting two-dimensional representation were annotated to known biological cell types using well-known marker genes.

CNA inference of single cells

The chromosomal CNA profile of single cells was inferred using the inferCNV (version 1.0.4) R package.[19] Immune cells from each patient were used as reference to define a baseline of normal karyotype such that their average copy number value was subtracted from all cells. To ensure accuracy, the following parameters were used: cutoff=0.1, noise_logistic=TRUE, sd_amplifier=1.5 and denoise=TRUE.

Epithelial score and epithelial-mesenchymal transition score

We used the expression levels of epithelial marker genes to confirm the classification of malignant and non-malignant cells. The epithelial score was calculated as the average expression level of a set of epithelial marker genes defined in this study.[20]

For patients with available scRNA-seq data, we used the same method as previously described [21] to calculate the EMT score for each single cell using the following formula:

$$\text{EMT score} = \sum Mesenchymal\ gene\ expressions - \sum Epithelial\ gene\ expressions$$

RNA-ITH based on the scRNA-seq data

We also calculated the RNA-ITH score for each patient with scRNA-seq data. Briefly, we first combined the normalized gene expression matrix of the malignant cells of all patients and kept genes that were expressed in more than 70% of cells. Next, we calculated the CV value for each gene across all cells of each patient. This process was repeated for all patients, yielding a patient-specific, gene-specific CV matrix. Then, for each patient, we selected the top 100 genes with the highest CV values and took the average as the RNA-ITH score for that specific patient.

Cell-cell interaction analysis

To enable a systematic analysis of cell-cell communication, we reclustered each cell type. The CellPhoneDB[22] package was then used to explore the interactions of ligand-receptor pairs between niche cell subtypes and malignant cells as previously reported [23]. The interactions between distinct cell subpopulations via putative ligand-receptor pairs were visualized using the ggplot2 package.

Gene set variation analysis

Gene set variation analysis (GSVA) was performed for all patients with available scRNA-seq data using the 50 hallmark gene signatures (version 7.2) obtained from the Molecular Signatures Database (https://www.gsea-msigdb.org/gsea/msigdb). All analyses were performed with default parameters using the gsva function as implemented in the GSVA R package (version 3.14.0).[24]

Expression programs of malignant cells

We applied NMF, as implemented in the NMF R packages (version 0.21.0), to extract transcriptional programs of malignant cells of each tumor. Briefly, for each tumor, we first excluded genes with SD < 0.5 and mean-centered the remaining genes across cells to obtain the relative expression values. Then we transformed the mean-centered expression matrix by replacing all negative values to zero. For each NMF run, we set the factors to 10 and for each of the resulting factors, we extracted the top 50 genes with the highest NMF scores as characteristics of that given factor. Each tumor was then scored according to these NMF programs. Hierarchical clustering of the scores for each program using Pearson correlation coefficients as the distance metric and the complete linkage revealed 3 correlated sets of meta-programs.

Stemness, cytotoxicity and exhaustion scores

We calculated the cell scores of certain gene sets by considering both average expression and the influence caused by different library complexities. As described by a previous study, we defined a control gene set in the calculation of different cell scores.[20] Briefly, we first partitioned all analyzed genes into 25 bins according to their average expression levels across all cells. Then, for each gene from the target gene set (Gt), we randomly selected 100 genes from the bin to which this gene belonged. These randomly selected genes comprised the control gene set (Gc). Finally, Cell scores (CS) were calculated as CS=average (Gt)-average (Gc).

Pseudotime trajectory analysis

To depict the developmental trajectory of $CD8^+$ T cells, the Monocle2 (version 2.12.0) R package was applied to the expression profiles of all $CD8^+$ T cells.[25] Briefly, the expression profiles (Seurat objects) were converted to Monocle cell data sets by the 'importCDS' function. Differentially expressed genes between $CD8^+$ T cells from *IDH*-SG samples and *IDH*-NO samples were tested using the 'differentialGeneTest' function. Putative trajectories were plotted using the 'plot_cell_trajectory' function and the pseudotime-dependent genes were visualized with the plot_pseudotime_heatmap function.

Plot generation

Boxplots were generated with the ggplot2 R package. The central rectangle spans the interquartile range (IQR, the first quartile to the third quartile), with the segment inside the rectangle corresponding to the median value. The lower and upper whiskers represent the minimum (or first quartile minus 1.5 x IQR) and the maximum (or third quartile plus 1.5 x IQR), respectively. Violin plots were generated with the ggplot2 R package. Kaplan-Meier plots of overall survival were generated with GraphPad Prism (version 7.0).

Hematoxylin-eosin staining and histological grading

The ICC tissues were fixed in 10% formalin and then embedded in paraffin wax blocks. Histological sections (4-5 μm thick) were prepared, dewaxed in xylene, rehydrated through decreasing concentrations of ethanol, and washed in PBS. The sections were stained with hematoxylin and eosin (H&E), after which they were dehydrated using increasing concentrations of ethanol and xylene.

Immunohistochemistry and immunofluorescence

Briefly, ICC patient tissues were fixed overnight with 10% neutral buffered formalin,

embedded in paraffin, and then processed as 4 μm sections. The sections were blocked in 20% donkey serum in PBS for 30 minutes and incubated with the following primary antibodies overnight in a humidified chamber: anti-HGF antibody (1:200, abcam, Cat# ab52625), anti-KRT7 antibody (1:300, abcam, ab52625), anti-KRT19 antibody (1:600, abcam, ab181598), anti-CD8 antibody (1:200, abcam, ab237709), and anti-PD1 antibody (1:400, abcam, ab52587).

Immunofluorescence

For immunofluorescence imaging of ICC tumors, whole tumors were fixed in 4% PBS overnight and dehydrated in 30% sucrose/PBS prior to embedding in OCT freezing medium. 10 mm sections were cut on a cryostat, adhered to Superfrost Plus slides (ThermoFisher) and permeabilized/blocked with PBS with 0.3% Triton X-100 (Sigma) and 10% goat serum (Jackson Immunoresearch). Sections were stained with directly conjugated antibodies (anti-CD8 antibody (1:200, abcam, ab237709); anti-GZMB antibody (1:300, abcam, ab25598)) in PBS with 0.1% Triton X-100 and 5% goat serum.

Follow-up

Patients were screened for carcinoembryonic antigen, CA199, and subjected to a computed tomography scan every 1-2 months for the first 6 months after surgery and every 3 months afterwards. When recurrence was suspected, magnetic resonance imaging or positron-emission tomography images were taken for confirmation. Disease-free survival was measured from the date of surgery to the date of recurrence. Follow-up of patients was continued until recurrence or June 2020.

Statistical analysis

Statistical analyses were performed using Graphpad Prism (version 7.0) and Rstudio (version 3.5.1). Comparisons between groups were conducted using the Chi-square

test or Fisher’s exact test for categorical variables and Student’s *t* test or the Wilcoxon rank-sum test for continuous variables. A value of $P < 0.05$ was considered statistically significant.

References

[1] Martin, *EMBnet* **2011**, *17*, 10, https://doi.org/10.14806/ej.17.1.200.

[2] H. Li, R. Durbin, *Bioinformatics* **2009**, *25* (14), 1754, https://doi.org/10.1093/bioinformatics/btp324.

[3] A. McKenna, M. Hanna, E. Banks, A. Sivachenko, K. Cibulskis, A. Kernytsky, K. Garimella, D. Altshuler, S. Gabriel, M. Daly, M. A. DePristo, *Genome Res* **2010**, *20* (9), 1297, https://doi.org/10.1101/gr.107524.110.

[4] K. Cibulskis, M. S. Lawrence, S. L. Carter, A. Sivachenko, D. Jaffe, C. Sougnez, S. Gabriel, M. Meyerson, E. S. Lander, G. Getz, *Nat Biotechnol* **2013**, *31* (3), 213, https://doi.org/10.1038/nbt.2514.

[5] S. Kim, K. Scheffler, A. L. Halpern, M. A. Bekritsky, E. Noh, M. Kallberg, X. Chen, Y. Kim, D. Beyter, P. Krusche, C. T. Saunders, *Nat Methods* **2018**, *15* (8), 591, https://doi.org/10.1038/s41592-018-0051-x.

[6] P. Cingolani, A. Platts, L. Wang le, M. Coon, T. Nguyen, L. Wang, S. J. Land, X. Lu, D. M. Ruden, *Fly (Austin)* **2012**, *6* (2), 80, https://doi.org/10.4161/fly.19695.

[7] F. Favero, T. Joshi, A. M. Marquard, N. J. Birkbak, M. Krzystanek, Q. Li, Z. Szallasi, A. C. Eklund, *Ann Oncol* **2015**, *26* (1), 64, https://doi.org/10.1093/annonc/mdu479.

[8] C. H. Mermel, S. E. Schumacher, B. Hill, M. L. Meyerson, R. Beroukhim, G. Getz, *Genome Biol* **2011**, *12* (4), R41, https://doi.org/10.1186/gb-2011-12-4-r41.

[9] A. Roth, J. Khattra, D. Yap, A. Wan, E. Laks, J. Biele, G. Ha, S. Aparicio, A. Bouchard-Cote, S. P. Shah, *Nat Methods* **2014**, *11* (4), 396, https://doi.org/10.1038/nmeth.2883.

[10] C. Zhang, L. Zhang, T. Xu, R. Xue, L. Yu, Y. Zhu, Y. Wu, Q. Zhang, D. Li, S. Shen, D. Tan, F. Bai, H. Zhang, *Nat Commun* **2020**, *11* (1), 1993, https://doi.org/10.1038/s41467-020-15886-6.

[11] K. Tamura, D. Peterson, N. Peterson, G. Stecher, M. Nei, S. Kumar, *Mol Biol*

Evol **2011**, *28* (10), 2731, https://doi.org/10.1093/molbev/msr121.

[12] A. Dobin, C. A. Davis, F. Schlesinger, J. Drenkow, C. Zaleski, S. Jha, P. Batut, M. Chaisson, T. R. Gingeras, *Bioinformatics* **2013**, *29* (1), 15, https://doi.org/10.1093/bioinformatics/bts635.

[13] M. I. Love, W. Huber, S. Anders, *Genome Biol* **2014**, *15* (12), 550, https://doi.org/10.1186/s13059-014-0550-8.

[14] D. Biswas, N. J. Birkbak, R. Rosenthal, C. T. Hiley, E. L. Lim, K. Papp, S. Boeing, M. Krzystanek, D. Djureinovic, L. La Fleur, M. Greco, B. Dome, J. Fillinger, H. Brunnstrom, Y. Wu, D. A. Moore, M. Skrzypski, C. Abbosh, K. Litchfield, M. Al Bakir, T. B. K. Watkins, S. Veeriah, G. A. Wilson, M. Jamal-Hanjani, J. Moldvay, J. Botling, A. M. Chinnaiyan, P. Micke, A. Hackshaw, J. Bartek, I. Csabai, Z. Szallasi, J. Herrero, N. McGranahan, C. Swanton, T. R. Consortium, *Nat Med* **2019**, *25* (10), 1540, https://doi.org/10.1038/s41591-019-0595-z.

[15] Y. C. Shen, C. L. Hsu, Y. M. Jeng, M. C. Ho, C. M. Ho, C. P. Yeh, C. Y. Yeh, M. C. Hsu, R. H. Hu, A. L. Cheng, *J Hepatol* **2020**, *72* (3), 489, https://doi.org/10.1016/j.jhep.2019.09.032.

[16] P. H. D. Nguyen, S. Ma, C. Z. J. Phua, N. A. Kaya, H. L. H. Lai, C. J. Lim, J. Q. Lim, M. Wasser, L. Lai, W. L. Tam, T. K. H. Lim, W. K. Wan, T. Loh, W. Q. Leow, Y. H. Pang, C. Y. Chan, S. Y. Lee, P. C. Cheow, H. C. Toh, F. Ginhoux, S. Iyer, A. W. C. Kow, Y. Young Dan, A. Chung, B. K. P. Goh, S. Albani, P. K. H. Chow, W. Zhai, V. Chew, *Nat Commun* **2021**, *12* (1), 227, https://doi.org/10.1038/s41467-020-20171-7.

[17] D. Aran, Z. Hu, A. J. Butte, *Genome Biol* **2017**, *18* (1), 220, https://doi.org/10.1186/s13059-017-1349-1.

[18] T. Stuart, A. Butler, P. Hoffman, C. Hafemeister, E. Papalexi, W. M. Mauck, 3rd, Y. Hao, M. Stoeckius, P. Smibert, R. Satija, *Cell* **2019**, *177* (7), 1888, https://doi.org/10.1016/j.cell.2019.05.031.

[19] I. Tirosh, B. Izar, S. M. Prakadan, M. H. Wadsworth, D. Treacy, J. J. Trombetta, A. Rotem, C. Rodman, C. Lian, G. Murphy, M. Fallahi-Sichani, K. Dutton-Regester, J. R. Lin, O. Cohen, P. Shah, D. Lu, A. S. Genshaft, T. K. Hughes, C. G. K. Ziegler, S. W. Kazer, A. Gaillard, K. E. Kolb, A. C. Villani, C. M. Johannessen, A. Y. Andreev, E. M. Van Allen, M. Bertagnolli, P. K. Sorger, R. J. Sullivan, K. T. Flaherty, D. T. Frederick, J. Jané-Valbuena, C. H. Yoon, O. Rozenblatt-Rosen, A. K. Shalek, A. Regev, L. A. Garraway, *Science* **2016**, *352* (6282), 189, https://doi.org/10.1126/science.aad0501.

[20] S. V. Puram, I. Tirosh, A. S. Parikh, A. P. Patel, K. Yizhak, S. Gillespie, C. Rodman, C. L. Luo, E. A. Mroz, K. S. Emerick, D. G. Deschler, M. A. Varvares, R.

Mylvaganam, O. Rozenblatt-Rosen, J. W. Rocco, W. C. Faquin, D. T. Lin, A. Regev, B. E. Bernstein, *Cell* **2017**, *171* (7), 1611, https://doi.org/10.1016/j.cell.2017.10.044.

[21] D. L. Gibbons, C. J. Creighton, *Dev Dyn* **2018**, *247* (3), 555, https://doi.org/10.1002/dvdy.24485.

[22] M. Efremova, M. Vento-Tormo, S. A. Teichmann, R. Vento-Tormo, *Nat Protoc* **2020**, *15* (4), 1484, https://doi.org/10.1038/s41596-020-0292-x.

[23] S. Jin, R. Li, M. Y. Chen, C. Yu, L. Q. Tang, Y. M. Liu, J. P. Li, Y. N. Liu, Y. L. Luo, Y. Zhao, Y. Zhang, T. L. Xia, S. X. Liu, Q. Liu, G. N. Wang, R. You, J. Y. Peng, J. Li, F. Han, J. Wang, Q. Y. Chen, L. Zhang, H. Q. Mai, B. E. Gewurz, B. Zhao, L. S. Young, Q. Zhong, F. Bai, M. S. Zeng, *Cell Res* **2020**, https://doi.org/10.1038/s41422-020-00402-8.

[24] A. Subramanian, P. Tamayo, V. K. Mootha, S. Mukherjee, B. L. Ebert, M. A. Gillette, A. Paulovich, S. L. Pomeroy, T. R. Golub, E. S. Lander, J. P. Mesirov, *Proc Natl Acad Sci U S A* **2005**, *102* (43), 15545, https://doi.org/10.1073/pnas.0506580102.

[25] C. Trapnell, D. Cacchiarelli, J. Grimsby, P. Pokharel, S. Li, M. Morse, N. J. Lennon, K. J. Livak, T. S. Mikkelsen, J. L. Rinn, *Nat Biotechnol* **2014**, *32* (4), 381, https://doi.org/10.1038/nbt.2859.

Supplemental figures

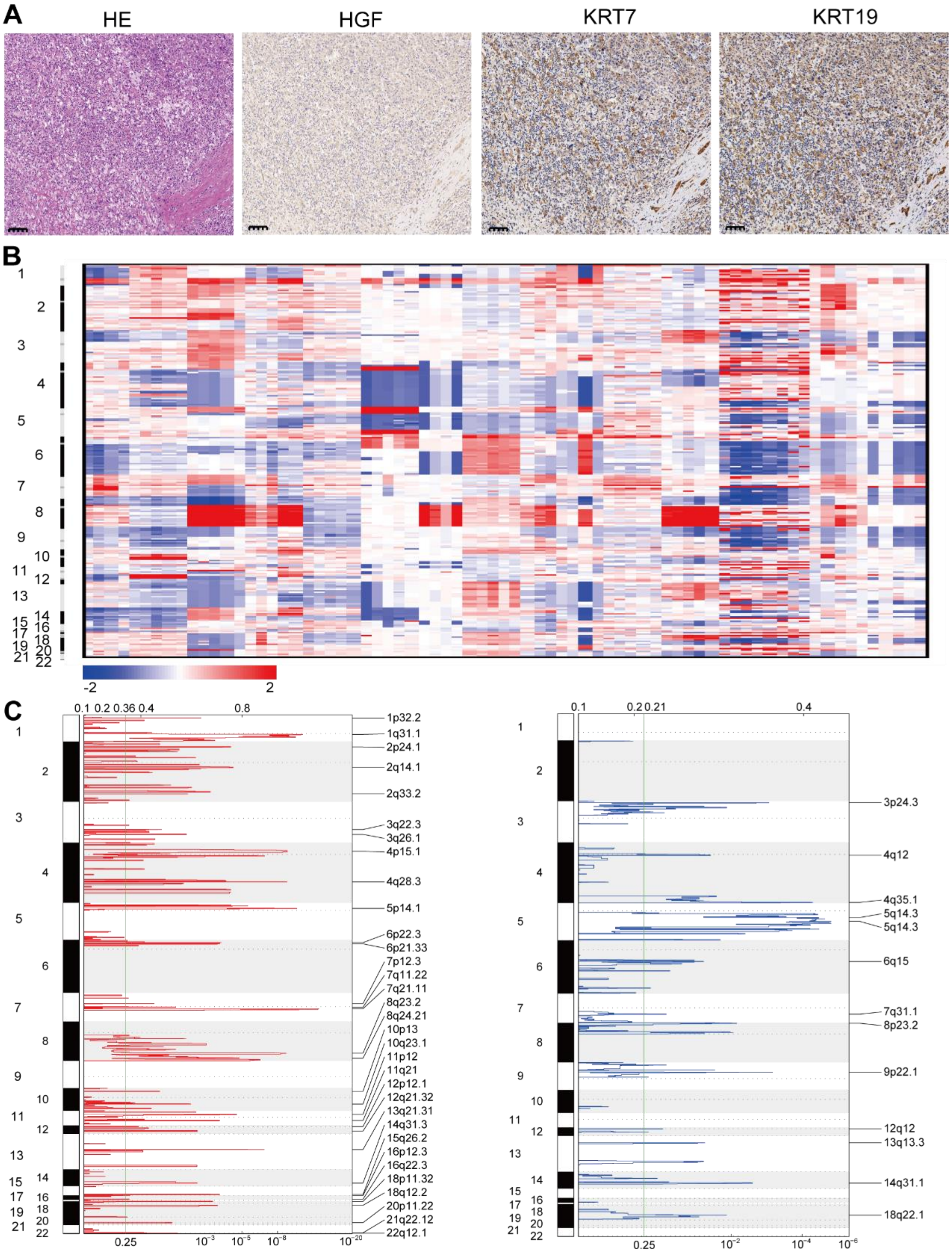

Figure S1. Pathology and copy number alterations of ICC tumors.

(A) Pathology of a representative ICC tumor sample. HE, hematoxylin and eosin; HGF, hepatocyte growth factor; KRT7, keratin 7; KRT19, keratin 19. Scale bar, 100

μm.

(B) Heatmap of CNAs. The Y-axis shows chromosomal coordinates.

(C) Significant CNAs in genomic regions detected by GISTIC 2.0. Altered regions are shown as peaks. Copy number deletions and amplifications are shown in blue (left) and red (right), respectively. The vertical lines in each graph indicate the default *q* value of 0.25.

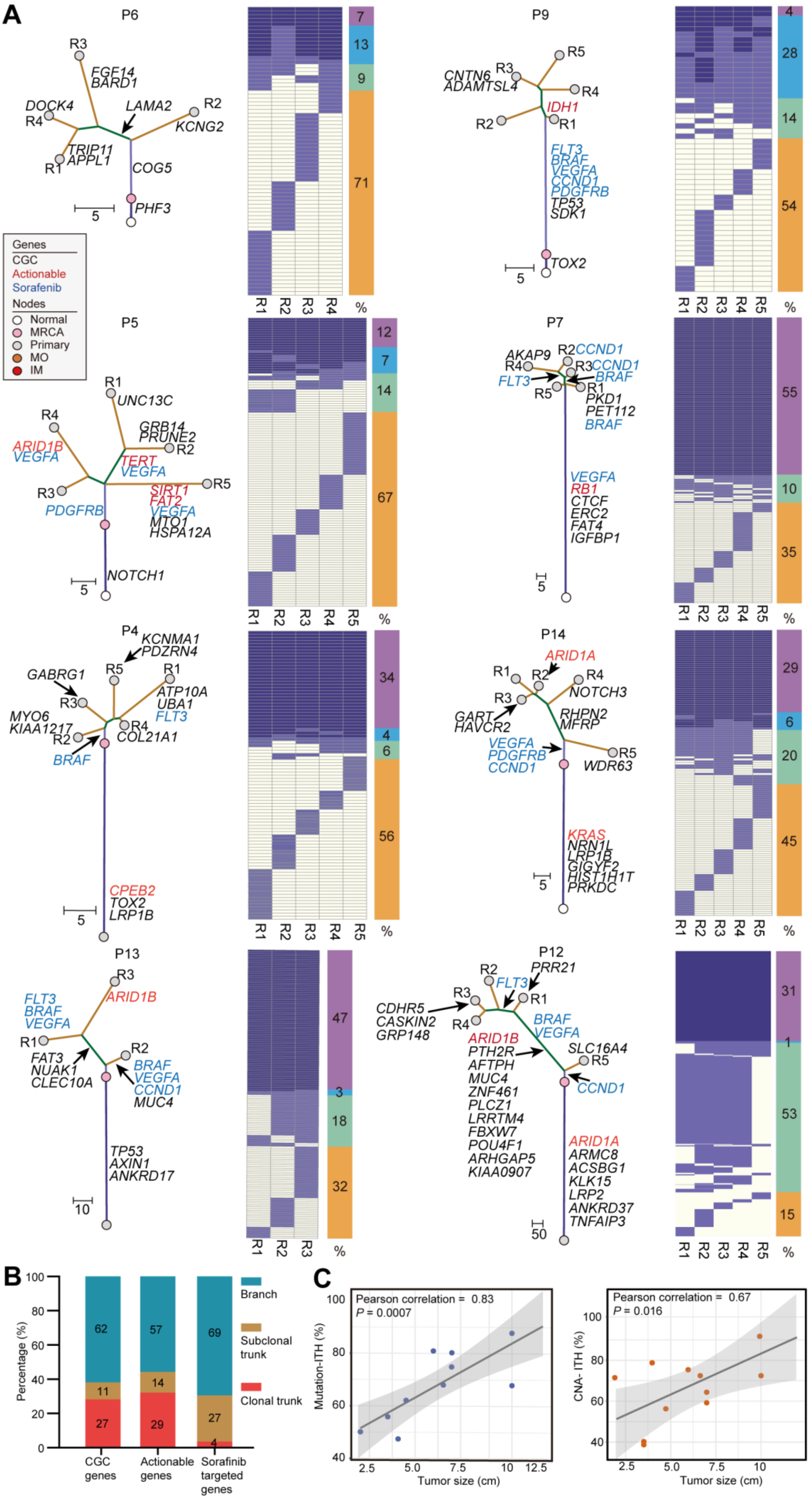
A
P6
R3
FGF14
BARD1
DOCK4
R4
LAMA2
R2
KCNG2
TRIP11
APPL1
R1
COG5
5
PHF3
7
13
9
71
R1 R2 R3 R4 %
Genes
CGC
Actionable
Sorafenib
Nodes
Normal
MRCA
Primary
MO
IM
P9
R5
CNTN6
ADAMTSL4
R3
R4
IDH1
R2
R1
FLT3
BRAF
VEGFA
CCND1
PDGFRB
TP53
SDK1
5
TOX2
4
28
14
54
R1 R2 R3 R4 R5 %
P5
R1
UNC13C
R4
GRB14
PRUNE2
R2
ARID1B
VEGFA
TERT
VEGFA
R3
SIRT1
FAT2
R5
PDGFRB
VEGFA
MTO1
HSPA12A
5
NOTCH1
12
7
14
67
R1 R2 R3 R4 R5 %
P7
AKAP9
R2
CCND1
R4
R3
CCND1
FLT3
BRAF
R5
R1
PKD1
PET112
BRAF
VEGFA
RB1
CTCF
ERC2
FAT4
IGFBP1
5
55
10
35
R1 R2 R3 R4 R5 %
P4
KCNMA1
PDZRN4
GABRG1
R5
R1
ATP10A
UBA1
R3
FLT3
MYO6
KIAA1217
R4
R2
COL21A1
BRAF
CPEB2
TOX2
LRP1B
5
34
4
6
56
R1 R2 R3 R4 R5 %
P14
ARID1A
R1
R2
R4
NOTCH3
R3
GART
HAVCR2
RHPN2
MFRP
VEGFA
PDGFRB
CCND1
R5
WDR63
KRAS
NRN1L
LRP1B
GIGYF2
HIST1H1T
PRKDC
5
29
6
20
45
R1 R2 R3 R4 R5 %
P13
R3
FLT3
BRAF
VEGFA
ARID1B
R1
R2
FAT3
NUAK1
CLEC10A
BRAF
VEGFA
CCND1
MUC4
TP53
AXIN1
ANKRD17
10
47
3
18
32
R1 R2 R3 %
P12
R2
PRR21
FLT3
R3
R1
CDHR5
CASKIN2
GRP148
R4
BRAF
VEGFA
ARID1B
PTH2R
AFTPH
MUC4
ZNF461
PLCZ1
LRRTM4
FBXW7
POU4F1
ARHGAP5
KIAA0907
SLC16A4
R5
CCND1
ARID1A
ARMC8
ACSBG1
KLK15
LRP2
ANKRD37
TNFAIP3
50
31
1
53
15
R1 R2 R3 R4 R5 %
B
Percentage (%)
100
80
60
40
20
0
62
11
27
57
14
29
69
27
4
CGC genes
Actionable genes
Sorafenib targeted genes
Branch
Subclonal trunk
Clonal trunk
C
Pearson correlation = 0.83
P = 0.0007
Mutation-ITH (%)
100
80
60
40
2.5
5.0
7.5
10
12.5
Tumor size (cm)
Pearson correlation = 0.67
P = 0.016
CNA- ITH (%)
100
80
60
40
2.5
5.0
7.5
10
Tumor size (cm)

Figure S2. Regional heatmap of somatic mutations in ICC tumors.

(A) Heatmaps show the regional distribution of all mutations in eight ICC patients. Clonal and subclonal mutations are marked in blue and light blue, respectively. The columns next to each heatmap show four categories of mutations and their percentages: trunk clonal mutations (purple); trunk subclonal mutations (sky blue); branch mutations (pale green); and region-specific mutations (orange). Phylogenetic trees were constructed using a maximum parsimony algorithm based on mutations identified in each patient. The length of each line is proportional to the number of mutations. Mutations in potential driver genes are indicated, including CGC genes (black) and druggable genes (red). Sorafenib-targeted amplifications are annotated in blue. Patient IDs and region names are labeled in each tree. MRCA, most recent ancestor, IM, intrahepatic metastasis, MO, multiple occurrences.

(B) The altered percentages of actionable genes and sorafenib-targeted genes affected by clonal trunk, subclonal trunk, and branch mutations.

(C) Scatter plots showing the correlations between the tumor size and the mutation-ITH (left) or CNA-ITH (right), respectively.

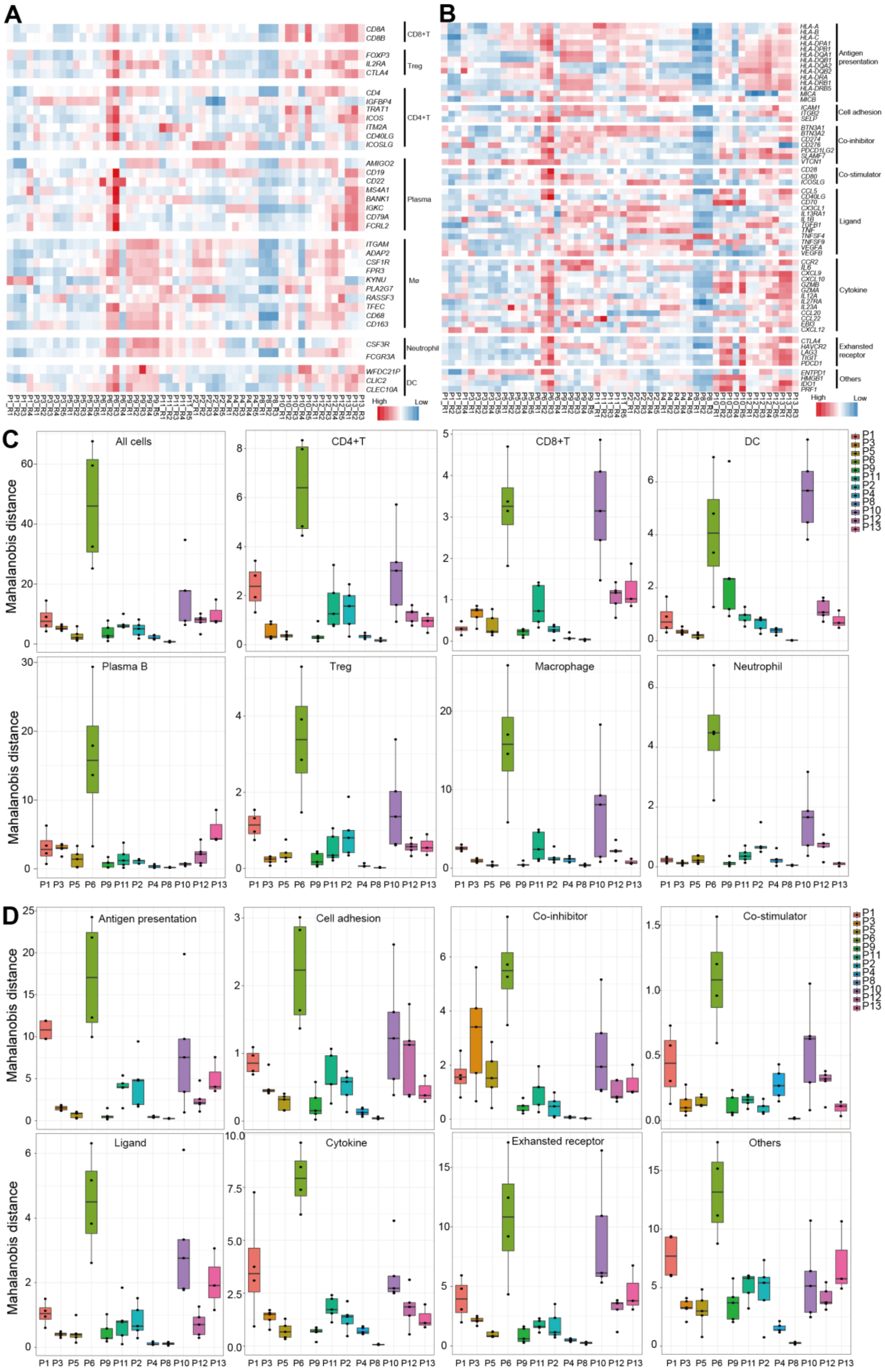

A
CD8A
CD8B
CD8+T
FOXP3
IL2RA
CTLA4
Treg
CD4
IGFBP4
TRAT1
ICOS
ITM2A
CD40LG
ICOSLG
CD4+T
AMIGO2
CD19
CD22
MS4A1
BANK1
IGKC
CD79A
FCRL2
Plasma
ITGAM
ADAP2
CSF1R
FPR3
KYNU
PLA2G7
RASSF3
TFEC
CD68
CD163
Me
CSF3R
FCGR3A
Neutrophil
WFDC21P
CLIC2
CLEC10A
DC
High
Low
B
HLA-A
HLA-B
HLA-C
HLA-DPA1
HLA-DPB1
HLA-DQA1
HLA-DQB1
HLA-DQA2
HLA-DQB2
HLA-DRA
HLA-DRB1
HLA-DRB5
MICA
MICB
Antigen presentation
ICAM1
ITGB2
SELP
Cell adhesion
BTN3A1
BTN3A2
CD274
CD276
PDCD1LG2
SLAMF7
VTCN1
Co-inhibitor
CD28
CD80
ICOSLG
Co-stimulator
CCL5
CD40LG
CD70
CX3CL1
IL13RA1
IL1B
TGFB1
TNF
TNFSF4
TNFSF9
VEGFA
VEGFB
Ligand
CCR2
IL6
CXCL9
CXCL10
GZMB
GZMA
IL12A
IL27RA
IL23A
CCL20
CCL22
EBI3
CXCL12
Cytokine
CTLA4
HAVCR2
LAG3
TIGIT
PDCD1
Exhansted receptor
ENTPD1
HMGB1
IDO1
PRF1
Others
High
Low
C
All cells
CD4+T
CD8+T
DC
Plasma B
Treg
Macrophage
Neutrophil
Mahalanobis distance
P1 P3 P5 P6 P9 P11 P2 P4 P8 P10 P12 P13
D
Antigen presentation
Cell adhesion
Co-inhibitor
Co-stimulator
Ligand
Cytokine
Exhansted receptor
Others
Mahalanobis distance
P1 P3 P5 P6 P9 P11 P2 P4 P8 P10 P12 P13

Figure S3. Heterogeneities in TMEs among different regions within a tumor.

(A) Heatmap of the expression of 35 immune cell markers.

(B) Heatmap of the expression of 63 immune-related functional markers.

(C) Mahalanobis distance based on 35 immune cell markers. Each dot denotes the immune cell density of a sample. The mean and standard deviation of the immune cell density in each tumor are shown. The first panel, labeled "All cells", shows the Mahalanobis distance plot when immunological markers of all immune cells ($CD8^+$ T, $CD4^+$ T, DC, Treg, macrophage, neutrophil, and plasma B cells) were considered. The other panels show the Mahalanobis distance plots when a single immunological marker was considered.

(D) Mahalanobis distance based on 63 immune-related functional markers.

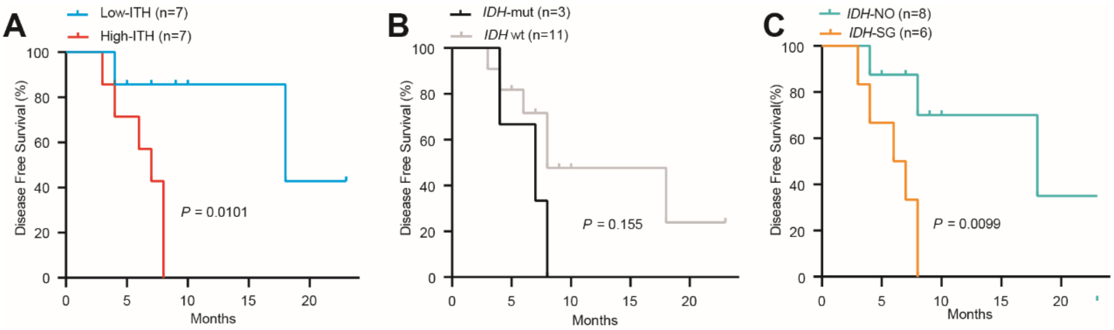

Figure S4. Recurrence analysis of 14 ICC tumors.

(A) Recurrence analysis of the high-ITH group and low-ITH group, *P* = 0.0101, log rank test.

(B) Recurrence analysis of *IDH*-mut and *IDH*-wt tumors, *P* = 0.155, log rank test.

(C) Recurrence analysis of *IDH*-SG and *IDH*-NO tumors, *P* = 0.0099, log rank test.

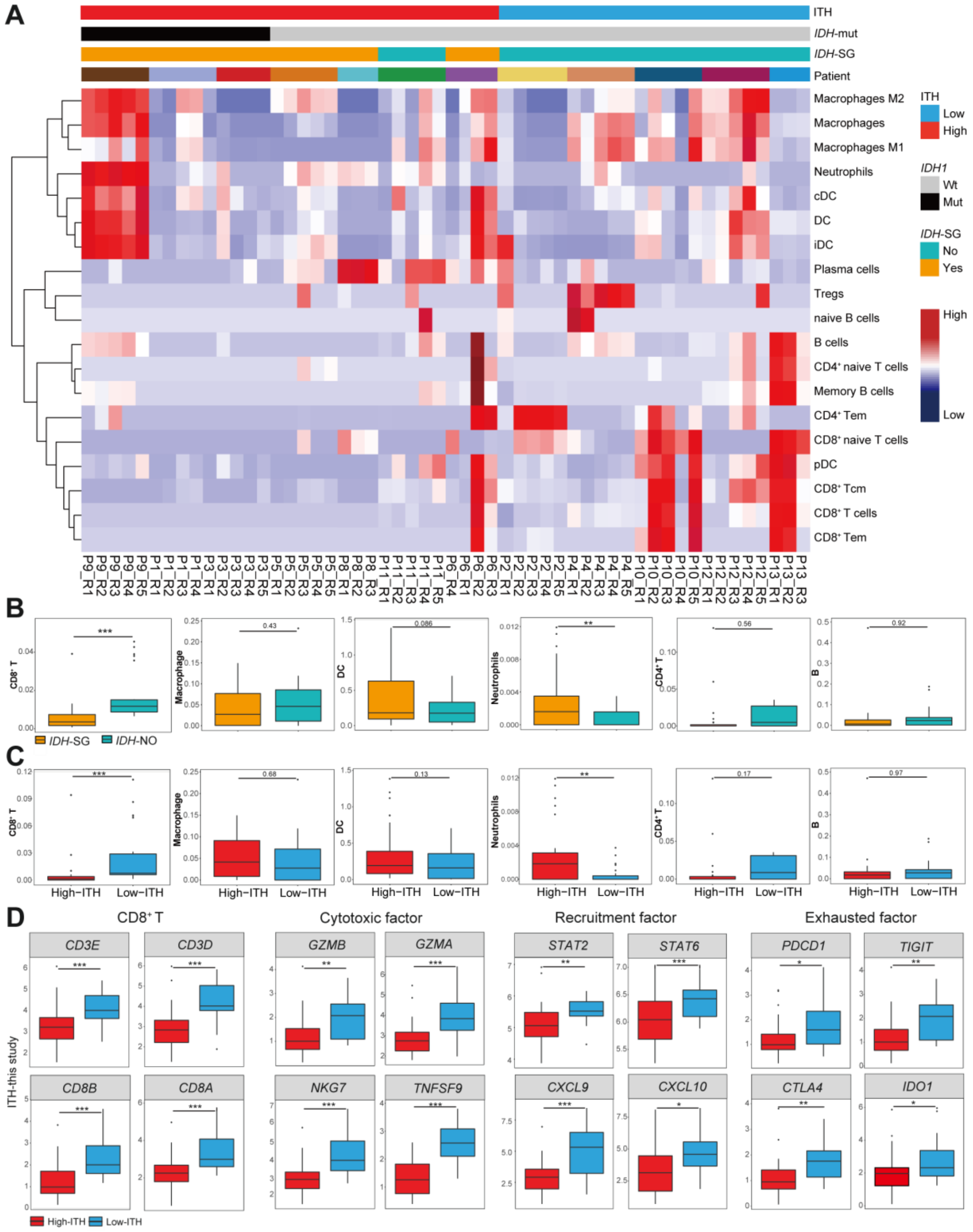

Figure S5. TME analysis of our ICC cohort.

(A) Composition of immune cells in ICC tumors using xCell.

(B) Comparison of immune cells between *IDH*-SG and *IDH*-NO patients. *: P <0.05, **: P <0.01, ***: P <0.001, Student's t test.

(C) Comparison of immune cells between high-ITH and low-ITH patients. *: P <0.05,

**: P <0.01, ***: P <0.001, Student's t test.

(D) Comparing the expression of $CD8^{+}$ T cell-related markers between high-ITH and low-ITH patients. *: P <0.05, **: P <0.01, ***: P <0.001, Student's t test.

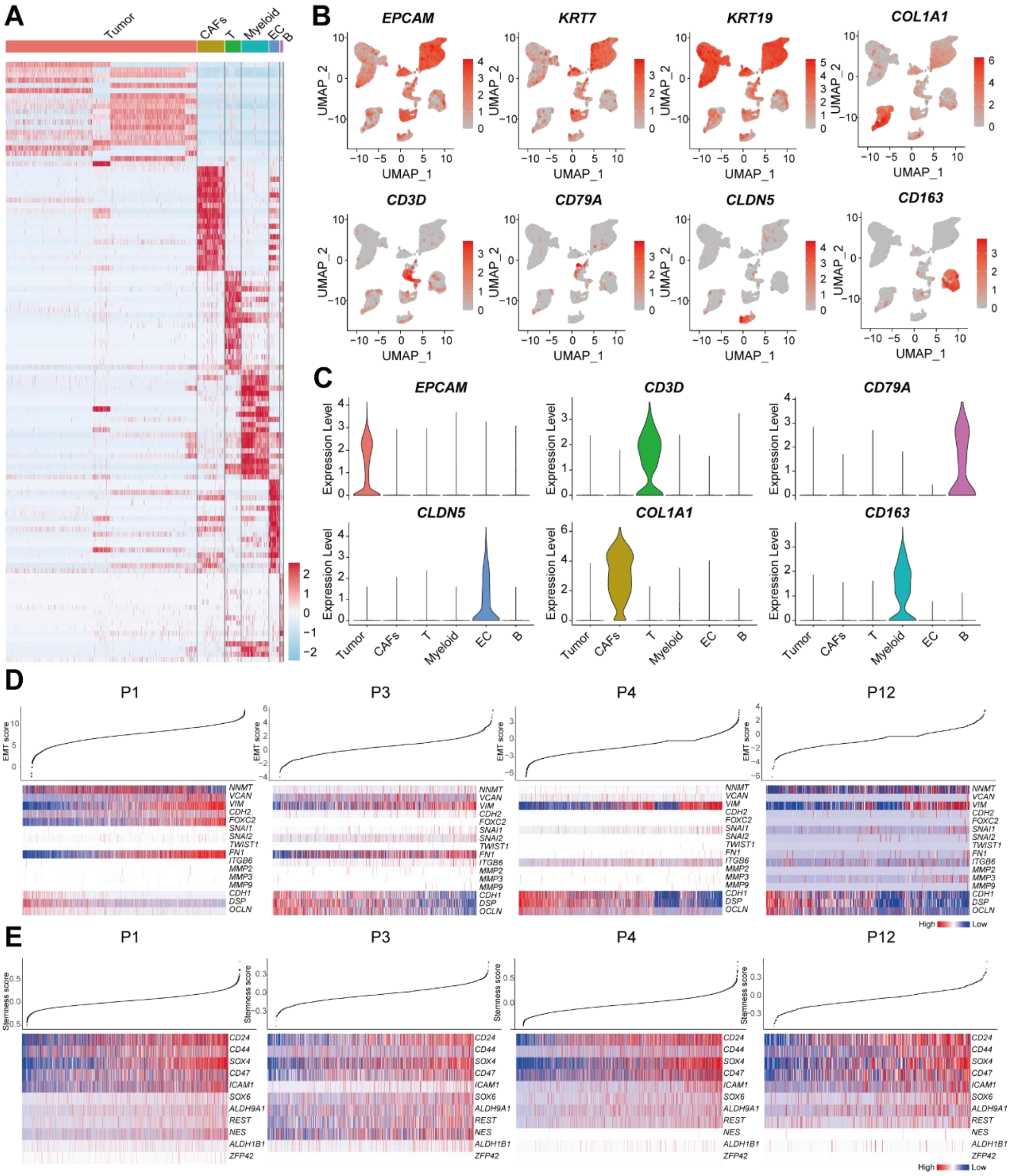

Figure S6. Single-cell analysis of *IDH*-SG and *IDH*-NO tumors.

(A) Heatmap showing the top DEGs in each cell type.

(B) t-SNE plots, color-coded for the expression (gray to red) of marker genes for each cell type, as indicated.

(C) Violin plots showing the expression of marker genes in distinct cell types.

(D) Each panel shows the EMT scores (top section of panel) and a heat map with expression of the 16 EMT related genes for four ICC tumors (bottom section of panel).

(E) Each panel shows the stemness scores (top section of panel) and a heat map with expression of the 11 stemness related genes for four ICC tumors (bottom section of panel).

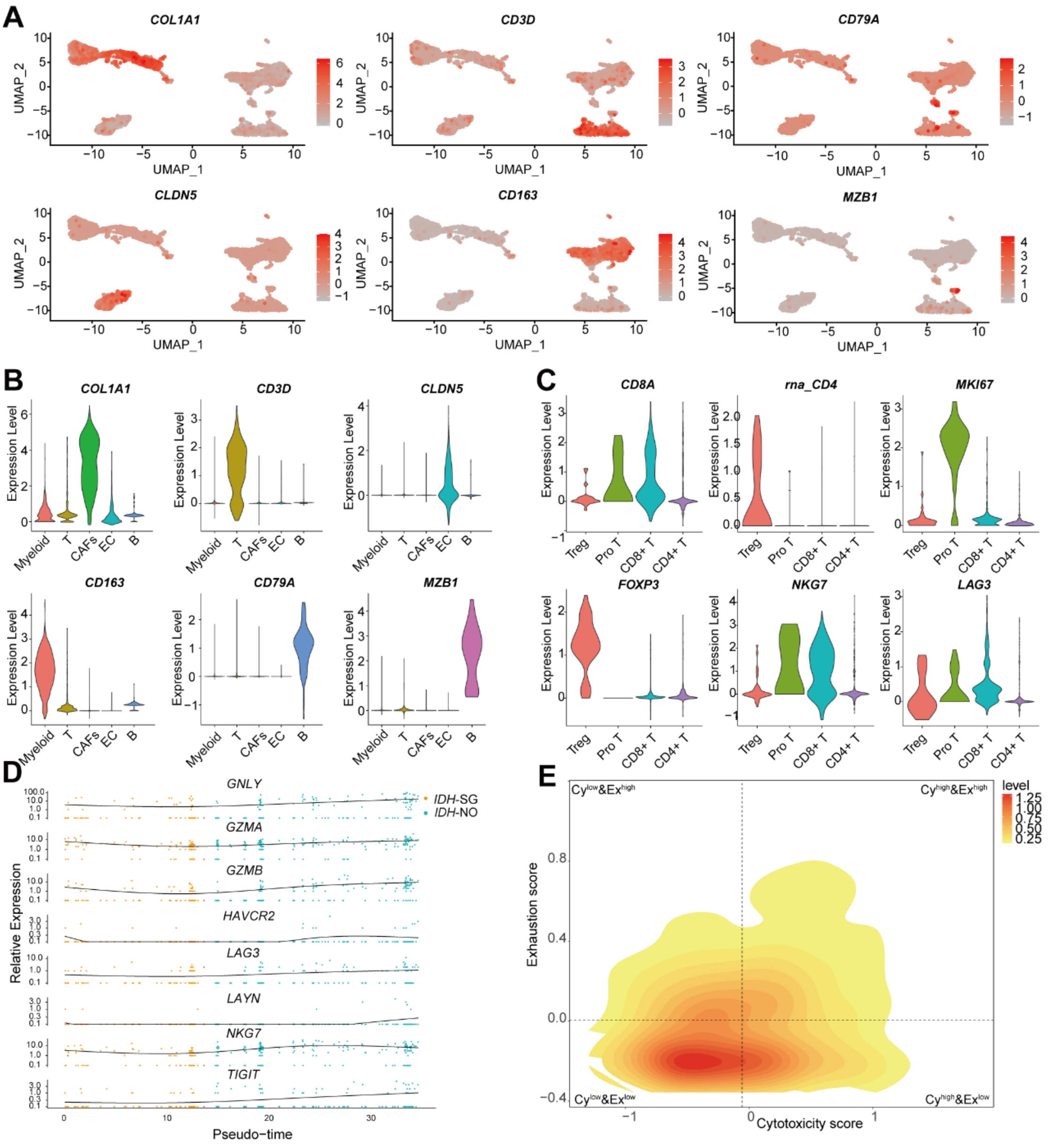

Figure S7. Single-cell analysis of non-malignant cells in *IDH*-SG and *IDH*-NO tumors.

(A) t-SNE plots, color-coded for the expression (gray to red) of marker genes for each non-malignant cell type, as indicated.

(B) Violin plots showing the expression of marker genes in distinct non-malignant cell types.

(C) Violin plots showing the expression of marker genes in distinct T cell types.

(D) Relative expression of *GNLY*, *GZMA*, *GZMB*, *HAVCR2*, *LAG3*, *LAYN*, *NKG7*,

and *TIGIT* along pseudotime.

(E) 2D density plot of the cytotoxicity and exhaustion states of $CD8^+$ T cells in four ICC tumors.
